# Biomechanics-guided design of polymeric membranes and thin films for smart medical devices

**DOI:** 10.3389/fbioe.2026.2011948

**Published:** 2026-10-26

**Authors:** Snehleen Kaur, Kartikeya Dubey, Gurpreet Singh, Vikramsingh Thakur

**Affiliations:** 1 Department of Materials Engineering, Indian Institute of Technology Jodhpur, Indra, Rajasthan, India; 2 Department of Materials Science and Engineering, Indian Institute of Technology Delhi, New Delhi, India; 3 School of Engineering and Materials Science, Queen Mary University of London, London, United Kingdom

**Keywords:** biointerfaces, biomechanics, biomedical thin films, polymeric membranes, smart medical devices, stimuli-responsive materials

## Abstract

Polymeric membranes and thin films are integral components of a wide range of medical devices, where their mechanical behaviour directly influences device function, durability, and interaction with surrounding tissues. Their design is challenging because physiological environments impose complex combinations of cyclic loading, deformation, fluid pressure, shear, viscoelasticity, and interfacial stresses. In this narrative review, we discuss a biomechanics-guided approach to designing polymeric membranes and thin films that link physiological mechanical requirements to polymer chemistry, material architecture, fabrication processes, interfacial behaviour, and device-level performance. We first examine the mechanical characteristics of representative biological tissues and discuss how these properties can be translated into material design requirements. We then review major polymer classes and manufacturing strategies, with particular emphasis on how molecular structure, crosslinking, fibre alignment, porosity, and multilayer architecture influence mechanical behaviour. The review further considers mechanics-transport coupling, stimuli-responsive membranes, fatigue and degradation under physiological conditions, and application-specific challenges in cardiovascular, neural, dialysis, and wound-care devices. Finally, emerging approaches based on machine learning, 4D printing, organ-on-chip systems, and biomimetic ultrathin membranes are discussed. We highlight that effective biomechanics-guided design requires consideration of not only elastic stiffness, but also anisotropy, viscoelasticity, fatigue, interfacial mechanics, degradation, and physiological conditioning. This framework provides a basis for integrating material and mechanical design considerations into the development of multifunctional polymeric membranes for medical applications.

## 1 Introduction

Polymeric membranes and thin films are increasingly used as mechanically active components of medical devices, including artificial organs, cardiovascular devices, neural interfaces, biosensors, drug-delivery systems, and wound-care platforms. Depending on the application, these materials may be exposed to cyclic deformation, pressure, shear, bending, fluid transport, and repeated interfacial loading (Stamatialis et al., 2008; Hasan et al., 2014). Consequently, their mechanical behaviour can directly influence device functionality, structural integrity, transport performance, and interactions with surrounding tissues. Conventional material selection for biomedical membranes has often focused on chemical stability, biocompatibility, processability, and functional performance. However, these criteria alone do not capture the mechanical demands imposed by physiological environments. A membrane that is mechanically inappropriate for its application may undergo excessive deformation, fatigue, creep, delamination, pore collapse, or stress concentration (Lacour et al., 2016; Guo et al., 2026a). Conversely, excessive mechanical stiffness may alter the local mechanical environment experienced by cells and tissues. Therefore, mechanical compatibility should be considered alongside biological and functional requirements in material and device design. We refer to this approach as biomechanics-guided design, in which the mechanical environment of the target tissue or device application is first defined and subsequently translated into material, structural, and manufacturing requirements (Holzapfel and Ogden, 2010; Fung, 2013). Importantly, mechanical compatibility should not be interpreted solely as matching Young’s modulus. Depending on the application, relevant properties may include tensile and bending stiffness, anisotropy, viscoelasticity, strength, fracture toughness, fatigue resistance, interfacial adhesion, and time-dependent degradation. Figure 1 shows the development of polymeric membranes in biomedical applications.

**FIGURE 1:**
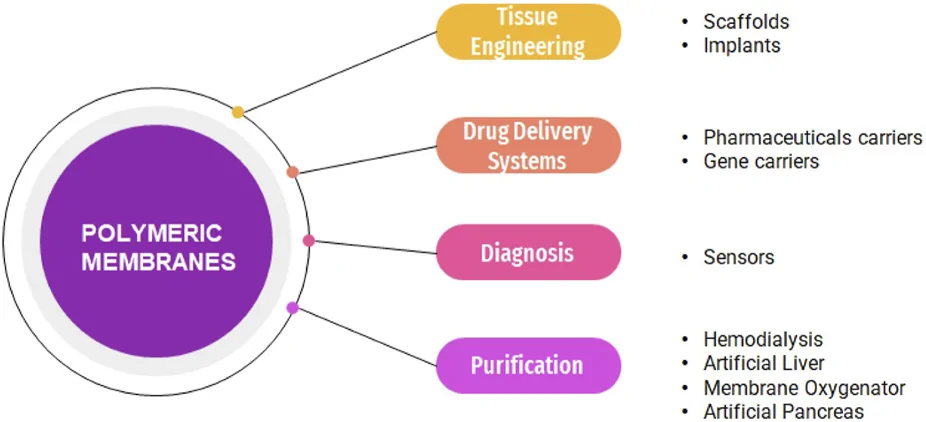
Polymeric membrane applications in the biomedical field. Reproduced under license CC-BY-4.0 (Radu et al., 2023).

Although numerous reviews have discussed biomedical polymers, smart membranes, flexible electronics, or individual fabrication technologies, an integrated framework connecting physiological mechanics, polymer structure, fabrication-induced architecture, interfacial behaviour, and device-level reliability remains useful for translational design. This narrative review, therefore, focuses on the mechanical principles that can guide the selection and engineering of polymeric membranes and thin films for smart medical devices. The review first considers the mechanical environment of representative biological tissues and translates these characteristics into material design requirements. It then discusses structure-property relationships in major polymer systems and examines how fabrication processes influence mechanical architecture. Subsequent sections address interfacial mechanics, mechanics-transport coupling, stimuli-responsive functionality, physiological degradation and fatigue, and application-specific examples. Finally, emerging approaches, including machine learning, 4D printing, organ-on-chip validation, and biomimetic ultrathin membranes, are discussed in the context of future biomechanics-guided design.

Conventional material selection for biomedical membranes often begins with intrinsic properties, such as elastic modulus, tensile strength, chemical stability, and biocompatibility, and seeks material that satisfies predefined performance criteria. Although these properties remain important, selecting a material primarily by matching its modulus to that of a target tissue may overlook the effects of loading mode, strain rate, cyclic deformation, viscoelasticity, interfacial stresses, and device geometry (Holzapfel and Ogden, 2010; Fung, 2013; Lacour et al., 2016; Guo et al., 2026b). Biomechanics-guided design extends this approach by beginning with the physiological and functional requirements of the intended application and translating them into material, structural, and manufacturing specifications. The key design logic is therefore iterative rather than based on a single material property. First, the relevant physiological loading conditions and functional requirements are defined. Second, candidate polymers are selected according to their mechanical, biological, and functional suitability. Third, membrane architecture, including thickness, porosity, fibre orientation, and multilayer configuration, and fabrication parameters are tailored to achieve the required mechanical response. Finally, the resulting design is evaluated under application-relevant mechanical and environmental conditions, and the findings are used to refine the material and structural design. Thus, biomechanics-guided design does not replace conventional material characterisation; it integrates material properties with physiological loading, structural architecture, fabrication, and device-level validation to support application-specific performance.

## 2 Physiological mechanical environment and design requirements

Polymeric membranes and thin films used in medical devices operate at the interface between engineered materials and dynamically biological tissues. Therefore, their design cannot be based solely on intrinsic polymer properties measured under standard laboratory conditions. The relevant design space is determined by the magnitude, direction, frequency, duration, and multiaxiality of physiological loading, together with the mechanical properties of the surrounding tissue and the presence of fluid, proteins, cells, and other environmental factors (Joodaki and Panzer, 2018b; Trombino et al., 2023a). A membrane intended for a cardiac, vascular, neural, cutaneous, or musculoskeletal application may therefore experience markedly different combinations of tensile, compressive, shear, bending, and cyclic loading (Muñoz Villaescusa et al., 2026).

This tissue-specific mechanical environment provides the first boundary condition for biomechanics-guided membrane design. Rather than seeking a universal target modulus, the objective is to identify the dominant mechanical functions of the intended application and translate them into appropriate requirements for stiffness, strength, extensibility, viscoelasticity, anisotropy, fatigue resistance, adhesion, and deformation compatibility. Importantly, mechanical matching should be considered together with membrane-specific requirements such as thickness, permeability, porosity, transport, and barrier function. The following sections, therefore, consider the physiological loading environment, the mechanical properties most relevant to membrane performance, and the translation of tissue-level mechanics into engineering design requirements.

### 2.1 Tissue-specific mechanical loading

For polymeric membranes, the most relevant parameters include stress and strain magnitude, strain rate, loading frequency, cyclic duration, pressure, shear, bending, and fluid-induced forces. These parameters determine not only the instantaneous deformation of the membrane but also its long-term fatigue, creep, interfacial stability, and functional performance. The cardiovascular system represents one of the most demanding environments for mechanically active polymeric membranes. Cardiac tissues undergo repeated deformation throughout the device’s lifetime, while blood-contacting surfaces are simultaneously exposed to pressure fluctuations and fluid-induced shear. The myocardium exhibits a relatively compliant, viscoelastic mechanical response, with reported tangential moduli ranging from tens to hundreds of kilopascals, depending on tissue region, strain level, loading direction, and measurement method. Literature studies report approximately 20–200 kPa at physiological strains of 15%–25%, together with substantial viscoelastic hysteresis associated with the collagen-rich extracellular matrix (Fatemifar et al., 2019; Wang et al., 2022). Such behaviour is particularly relevant to flexible cardiac interfaces because a membrane that is substantially stiffer than the surrounding tissue can locally restrict deformation and increase interfacial stresses. Conversely, insufficient stiffness or fatigue resistance may lead to excessive deformation, wrinkling, tearing, or loss of device geometry. Arterial tissues present a different combination of loading conditions. The arterial wall experiences cyclic circumferential stretch superimposed on blood-pressure-induced deformation and spatially varying fluid shear. Its nonlinear response arises from the progressive recruitment of collagen fibres, while elastin dominates the low-strain response. Holzapfel-Gasser-Ogden (HGO) hyperelastic constitutive model identifies an approximate circumferential stiffness range of 0.1–4 MPa (Holzapfel and Ogden, 2010), although this should be interpreted as a representative range rather than a universal material property because arterial mechanics depend strongly on anatomical location, age, pathology, loading direction, and experimental methodology. For vascular membranes, therefore, reproducing only the tensile modulus is insufficient; compliance, anisotropy, burst resistance, fatigue behaviour, and interaction with blood flow are all relevant.

The skin provides the principal mechanical environment for wearable films, epidermal sensors, flexible electrodes, and wound dressings. Skin undergoes repeated tensile deformation during movement and joint articulation, with mechanical behaviour strongly influenced by collagen fibre recruitment, hydration, anatomical location, age, and loading direction. Skin reports a low-strain modulus on the order of 0.1–0.5 MPa, with substantially higher apparent stiffness at larger strains as collagen fibres become increasingly recruited (Joodaki and Panzer, 2018a). This nonlinear behaviour has direct implications for wearable membranes: matching a single elastic modulus at one strain level does not guarantee mechanical compatibility across the full physiological strain range. Membrane designs should instead consider the strain amplitude experienced during daily movement, resistance to repeated stretching, conformability to curved surfaces, and adhesion under sweat and moisture exposure. At the opposite end of the compliance spectrum, the brain presents an extremely soft mechanical environment. Reported storage moduli of brain tissue range from approximately 0.5–2 kPa (Budday et al., 2015), although substantial variation exists between grey and white matter and across experimental techniques and loading frequencies. This extreme compliance creates a major challenge for implanted neural electrodes and protective encapsulation layers. Consequently, neural membranes require not only low effective stiffness but also high conformability, low bending rigidity, stable adhesion, and sufficient environmental protection. Soft polymeric systems such as PDMS, polyurethane-based elastomers, hydrogels, and ultrathin polymer films have therefore attracted considerable interest in mechanically compliant neural interfaces. Articular cartilage experiences compressive stresses in the MPa range, within a range of approximately 1–18 MPa, depending on the loading condition and anatomical site (Humphrey, 2013). Its behaviour is governed by the interaction between a porous extracellular matrix and interstitial fluid, meaning that load support and fluid transport cannot be considered independently. This provides an important analogy for membrane systems in which mechanical deformation modifies permeability or solute transport. In blood-contacting membranes, physiological wall shear stress varies substantially between vascular locations and flow states. Literature studies consider values on the order of 1–100 dyn/cm^2^ as relevant to blood-contacting membrane design (Davies, 2009; Badimon et al., 2012). Such shear is not merely a mechanical loading parameter. It can influence endothelial mechanotransduction, platelet activation, protein adsorption, and thrombotic behaviour. Accordingly, the mechanical design of a blood-contacting membrane must consider the coupled effects of wall deformation, fluid shear, surface chemistry, and haemocompatibility. These examples demonstrate that the physiological environment should be characterised by a mechanical loading profile rather than by a single tissue modulus.

### 2.2 Mechanical properties for membrane design

Translation of tissue mechanics into membrane design requires consideration of several complementary mechanical properties. Young’s modulus or stiffness is an important starting point because excessive stiffness mismatch can concentrate stresses at the tissue-device interface. However, modulus alone provides an incomplete description of membrane behaviour, particularly for soft, viscoelastic, anisotropic, or highly deformable tissues. The modulus of a membrane should be selected based on the mechanical role it serves (Lecomte et al., 2018). For load-bearing or pressure-containing membranes, sufficient stiffness may be required to maintain geometry and resist excessive deformation (Cho et al., 2021). In contrast, neural, cutaneous, and other soft-tissue interfaces generally require lower effective stiffness and bending rigidity to accommodate tissue motion. However, modulus matching should not be interpreted as simply selecting a polymer whose Young’s modulus equals that of the target tissue. Biological tissues are nonlinear, viscoelastic, anisotropic, hydrated, and often structurally heterogeneous. The appropriate target may instead be the membrane’s effective tensile, bending, or interfacial response under the specific loading mode encountered by the device (Mostafavi Yazdi and Baqersad, 2022; Avril et al., 2026). Strength and toughness are two properties during membrane design which primarily affect its performance and biomechanics. Strength defines the stress level that a membrane can withstand before yielding, rupture, or other irreversible failure, whereas toughness describes its capacity to absorb mechanical energy before failure. These properties become particularly important for thin films because defects, surface imperfections, pores, interfaces, and manufacturing-induced heterogeneities can act as stress concentrators. For highly extensible membranes, elongation at break alone can also be misleading. A membrane may tolerate large strains but possess insufficient tear resistance or fatigue life under repeated loading. Therefore, tensile strength should ideally be considered together with fracture toughness, tear resistance, crack-growth behaviour, and defect tolerance, particularly for long-term implantable applications (Niemiec and Kim, 2023).

Many tissues are structurally anisotropic because collagen or other fibrous components are preferentially aligned. Arterial walls, tendons, myocardium, and skin therefore exhibit direction-dependent mechanical behaviour. Membrane fabrication can similarly generate anisotropy (Annaidh et al., 2012; Sherman et al., 2015). Electrospinning, for example, can align fibres along a preferred direction, allowing the tensile response to be tailored independently in different directions. This provides an important design opportunity: rather than attempting to reproduce a tissue using an isotropic polymer, membrane architecture can be engineered to reproduce the dominant directional mechanical behaviour of the target tissue. For implanted and wearable devices, mechanical performance must be maintained over repeated physiological cycles. Fatigue resistance is therefore often more important than the ultimate tensile strength measured in a single monotonic test. Cardiac membranes, for example, may experience hundreds of millions of cycles over their service life, while neural, vascular, and wearable membranes can also undergo repeated deformation. Accordingly, fatigue testing should reproduce the frequency, strain amplitude, environment, and loading mode relevant to the application. For ultrathin films and flexible devices, bending stiffness can be more important than bulk tensile modulus. Bending stiffness decreases strongly with thickness, approximately scaling with the cube of membrane thickness for a homogeneous plate-like structure. Consequently, an ultrathin polymer film can remain highly conformable even when its bulk modulus is considerably greater than that of the surrounding tissue (Sun et al., 2015; Oroujzadeh et al., 2024; Sunwoo et al., 2024).

### 2.3 From tissue mechanics to material requirements

The central objective of biomechanics-guided design is to translate the physiological loading environment into measurable engineering specifications. It should be treated as a multivariable design problem in which mechanical loading, tissue properties, membrane geometry, material architecture, and functional requirements are considered simultaneously. For example, a cardiac leaflet membrane exposed to repetitive flexure requires a combination of appropriate compliance, anisotropy, fatigue resistance, tear resistance, haemocompatibility, and geometric stability (Wang et al., 2024c; Jalandhra et al., 2025). A neural membrane requires low bending stiffness, high conformability, stable electrical or optical performance, and resistance to moisture ingress. A wound dressing requires sufficient extensibility to follow skin motion while maintaining adhesion, controlled permeability, and potentially sensing or drug-delivery functions. A dialysis or filtration membrane must simultaneously withstand transmembrane pressure, fluid shear, repeated handling, and long-term exposure to protein-containing fluids while maintaining its transport properties (Cho et al., 2021; Bacal et al., 2024; Wang C. et al., 2024).

Increasing crosslink density may improve strength and dimensional stability but reduce extensibility and permeability. Increasing porosity may improve transport but reduce stiffness and fatigue resistance. Adding conductive or reinforcing nanofillers may improve electrical sensitivity or strength but may also introduce local stress concentrations, alter degradation behaviour, or reduce processability (Schmidt et al., 2025; Sharma et al., 2026). Similarly, increasing membrane thickness may improve mechanical durability but increase bending stiffness and reduce tissue conformity (Yamagishi et al., 2026). This perspective also highlights why mechanical properties measured under dry, ambient conditions are often insufficient for selecting biomedical membranes. Hydration, temperature, ionic strength, protein adsorption, cyclic loading, and degradation can substantially alter polymer stiffness, strength, permeability, and interfacial behaviour. The following sections subsequently address these effects through their discussions of polymer selection, transport-mechanics coupling, and physiological validation (Raghuwanshi and Garnier, 2019; Yamagishi et al., 2026). Table 1 shows the tissue-specific mechanical environments and corresponding design requirements for polymeric membranes in biomedical applications.

**TABLE 1:** Biomechanical characteristics of physiological tissues and membrane design considerations.

Physiological application	Dominant loading	Representative mechanical characteristics	Key membrane requirements	Example device
Cardiac	Cyclic tension, bending, pressure	Compliant, viscoelastic, anisotropic	Fatigue resistance, tear resistance, compliance, haemocompatibility	TAVR leaflet
Vascular	Circumferential stretch, pressure, shear	Nonlinear, anisotropic	Compliance, burst resistance, fatigue resistance, low thrombogenicity	Vascular membrane/graft
Neural	Low-amplitude strain, micromotion	Very soft, viscoelastic	Low bending stiffness, conformability, adhesion	Neural probe
Skin/wound	Tension, shear, bending	Nonlinear, strain-dependent	Extensibility, adhesion, permeability, fatigue resistance	Wearable sensor/wound dressing
Cartilage/joint	Compression, shear, fluid transport	Poroviscoelastic	Compression resistance, transport control, fatigue resistance	Cartilage interface
Blood-contacting membrane	Pressure + fluid shear	Fluid-structure coupled	Mechanical stability, permeability, haemocompatibility	Dialysis/oxygenation membrane

## 3 Polymer material selection: structure-property-biomechanics relationships

Selection of a polymer for a biomedical membrane should be guided by mechanical and functional requirements rather than by biocompatibility or processability alone. These properties can be further modified through membrane architecture and fabrication. No single polymer family satisfies all requirements across cardiac, neural, vascular, cutaneous, and drug-delivery applications. Instead, the appropriate material is determined by the required combination of mechanical compliance, fatigue resistance, environmental stability, permeability, electrical functionality, degradation profile, and manufacturability. Table 2 summarises representative polymer classes and their relevance to biomechanics-guided membrane design.

**TABLE 2:** Polymers for biomechanics-guided medical membrane design.

Polymer class	Representative systems	Biomechanically relevant properties	Functional advantages	Key limitations	Representative applications	Refs.
PU/PCU	Pellethane, Tecoflex, Bionate	Tunable stiffness, high extensibility, fatigue resistance	Toughness, flexibility	Oxidative degradation, ESC	Cardiac, VAD, wound	(Ji et al., 2024; Yi et al., 2026)
PDMS/silicones	Medical-grade PDMS	Low modulus, high extensibility	Flexibility, transparency	Fatigue, permeability/bleed concerns	Neural, retinal, flexible sensors	(Vėbraitė and Hanein, 2021)
PLA/PLGA/PCL	PLGA, PLLA, PCL	High initial stiffness; degradation-dependent	Resorbability	Property loss during degradation	Scaffolds, drug delivery	(Manavitehrani et al., 2016)
Hydrogels	PVA, GelMA, PNIPAM	Very low stiffness, viscoelastic	Tissue-like hydration, responsiveness	Tear resistance, dehydration	Neural, cartilage, wound	(Lu et al., 2024)
PVDF/ePTFE	PVDF-TrFE, ePTFE	Functional anisotropy, electroactivity	Piezoelectric sensing, vascular functionality	Delamination, calcification	Sensors, vascular membranes	(Zizhou et al., 2022)
Polyimide/Parylene	PI, Parylene-C	High strength and dimensional stability	Barrier/electrical insulation	High stiffness, adhesion	Neural electronics	(Liu et al., 2021)
Conductive composites	PDMS/CNT, PDMS/MXene, PEDOT:PSS	Tunable mechanics + conductivity	Sensing and bioelectronics	Percolation instability	E-skin, ECG, strain sensors	(Seyedin et al., 2014)

### 3.1 Polyurethanes and polycarbonate urethanes

Polyurethanes (PUs) are particularly attractive for mechanically demanding medical membranes because their segmented structure permits broad control of stiffness, elasticity, toughness, and degradation behaviour. Variation in hard-segment content, soft-segment chemistry, molecular weight, and crosslinking can produce materials spanning a wide mechanical range (Zdrahala and Zdrahala, 1999). This tunability makes PU-based systems suitable for applications that require repeated deformation, including cardiac membranes, vascular devices, wound dressings, and ventricular assist device components. On the other hand, polycarbonate urethanes (PCUs), such as Bionate® and Carbothane®, are particularly relevant for long-term implantable applications because they offer improved resistance to oxidative degradation compared with certain polyether-based polyurethane systems. This is important where cyclic loading occurs in the presence of physiological fluids and reactive species (Gunatillake et al., 2003). Recent research on bioderived electrospun membranes made from thermoplastic polyurethane (TPU) and polylactic acid (PLA) has demonstrated significant mechanical strength, with an ultimate tensile strength (UTS) greater than 15 MPa and elongation exceeding 300%. This biopolymer blending also offers the advantage of controlled biodegradability (Chambers et al., 2024). Additionally, electrospun polycarboxylic urethane (PCU) meshes coated with polyethylene glycol (PEG) hydrogel, designed for heart valve leaflet applications, exhibit anisotropic elastic behaviour. Specifically, the circumferential modulus is approximately 1.5 MPa, while the radial modulus is around 0.4 MPa, effectively mimicking the mechanical response of native aortic valve cusps (Pilecco et al., 2021). From a biomechanics perspective, the principal advantage of PU/PCU is therefore not simply high strength or extensibility, but the ability to independently tune mechanical compliance and durability over clinically relevant strain ranges.

At the molecular level, polyurethane mechanics are governed by the interplay between hard- and soft-segment chemistry, segmental organisation, molecular weight, and the extent of physical or chemical crosslinking. Hard-segment domains can act as reinforcing physical junctions, whereas the soft segments provide chain mobility and extensibility. Changes in segment composition and phase separation therefore influence stiffness, tensile strength, hysteresis, and energy dissipation under cyclic loading. For membrane applications, these effects should be evaluated together with fatigue resistance and environmental stability, because a composition that provides high initial strength may not necessarily retain its mechanical performance during prolonged physiological loading.

### 3.2 Silicone and PDMS-based materials

Polydimethylsiloxane (PDMS) and related silicone elastomers exhibit extreme flexibility, optical transparency, chemical stability, and low stiffness, all of which are required. Their relatively low modulus and high extensibility make them attractive for neural interfaces, flexible electronics, retinal and cochlear devices, and soft biomedical sensors. Crosslink density and network structure can be adjusted to modify stiffness and deformation behaviour. PDMS can also serve as a matrix for conductive fillers such as carbon nanotubes, graphene, and MXenes. Such composites provide a route toward mechanically compliant membranes that simultaneously exhibit strain-sensitive electrical behaviour. However, increasing filler content can alter elasticity, fatigue behaviour, and percolation stability. Long-term implant applications must also consider issues such as low-molecular-weight species, surface modification, moisture exposure, and interfacial adhesion (Chortos et al., 2016). Thus, PDMS-based membranes are particularly suited to applications where mechanical compliance and functional integration are more important than high load-bearing capacity (see Figure 2).

**FIGURE 2:**
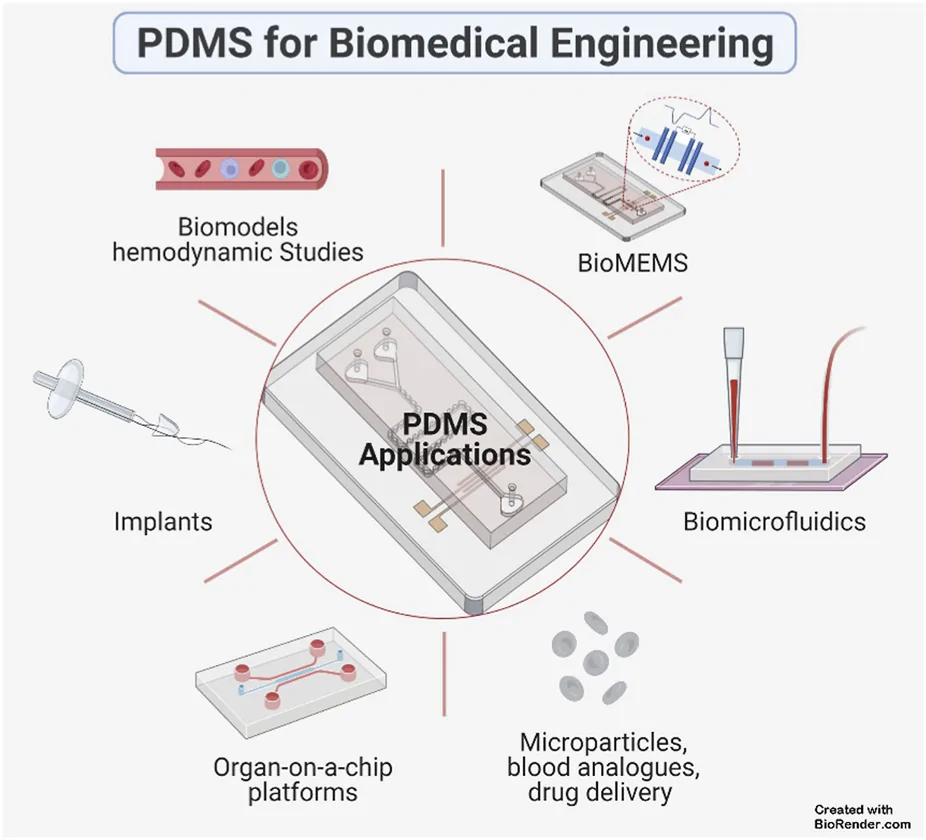
Applications of PDMS in biomedical engineering. Reproduced under license CC-BY-4.0 (Miranda et al., 2022).

### 3.3 Biodegradable polyesters

The primary biodegradable polyesters utilised in resorbable membrane applications, particularly for tissue-engineering scaffolds and transient drug delivery barriers, are poly (lactic acid) (PLA), poly (lactic-co-glycolic acid) (PLGA), and polycaprolactone (PCL). Their hydrolytic degradation kinetics can be adjusted by factors such as monomer ratio (e.g., PLGA 50:50 versus 75:25), molecular weight, and crystallinity. It is crucial for these kinetics to align with the remodelling dynamics of the host tissue to prevent the occurrence of a mechanical vacuum, the critical phase when the scaffold’s load-bearing capacity has decreased before the newly formed tissue has developed sufficient mechanical strength (Nair and Laurencin, 2007). Degradation is consistently associated with a decline in mechanical properties; for instance, the tensile strength of PLGA membranes can diminish by 50%–80% following 4–8 weeks of immersion in aqueous media. Consequently, employing accelerated ageing protocols under physiologically relevant conditions, such as using phosphate-buffered saline at 37 °C, is a crucial step in validating the design.

The influence of molecular weight and crystallinity extends beyond degradation rate to the retention of membrane mechanics during resorption. Chain scission reduces the molecular weight of degradable polyesters and may progressively compromise strength, ductility, and resistance to crack propagation. Crystalline regions can influence water penetration and degradation behaviour, while the resulting changes in morphology may alter stiffness, brittleness, and dimensional stability over time. Consequently, biodegradable membrane design should consider the evolution of mechanical properties throughout degradation, rather than relying exclusively on initial tensile strength or a nominal degradation period.

### 3.4 Hydrogels and hydrated polymer networks

Hydrogels are polymer networks that are saturated with water, making them the closest mechanical analogs to ultrasoft biological tissues, such as brain matter, cartilage, and vitreous humor. Their equilibrium moduli can be finely tuned within the range of 0.005–100 kPa by adjusting factors such as cross-link density, polymer concentration, and interpenetrating network (IPN) architecture (Annabi et al., 2014). Leading materials for neural interfaces, cartilage repair membranes, and smart wound dressings include GelMA, PVA double-network hydrogels, and PNIPAM-based systems. Research by Chen et al. demonstrated that a gelatin-based metamaterial hydrogel film, capable of achieving a tunable elastic modulus from 20 to 420 kPa and a Poisson’s ratio from −0.25 to 0.52, can effectively mimic the mechanical properties of myocardial and pulmonary tissues (Chen et al., 2024). This capability allows for advanced monitoring of cardiac deformation in implantable applications. Furthermore, conductive hydrogels incorporating PEDOT:PSS or MXene can simultaneously achieve tissue-mimetic mechanics and the electrical conductivity required for bioelectronic interface applications (Zhou et al., 2023). Because hydrogel mechanics are strongly dependent on water content, polymer concentration, crosslink density, ionic environment, and testing rate, reported modulus values should be compared only under comparable testing conditions. Their principal advantage is the ability to achieve very low effective stiffness while maintaining high water content, making them relevant to neural interfaces, cartilage-related applications, wound dressings, and soft bioelectronics. Figure 3 shows the applications of hydrogel membranes in various biomedical fields.

**FIGURE 3:**
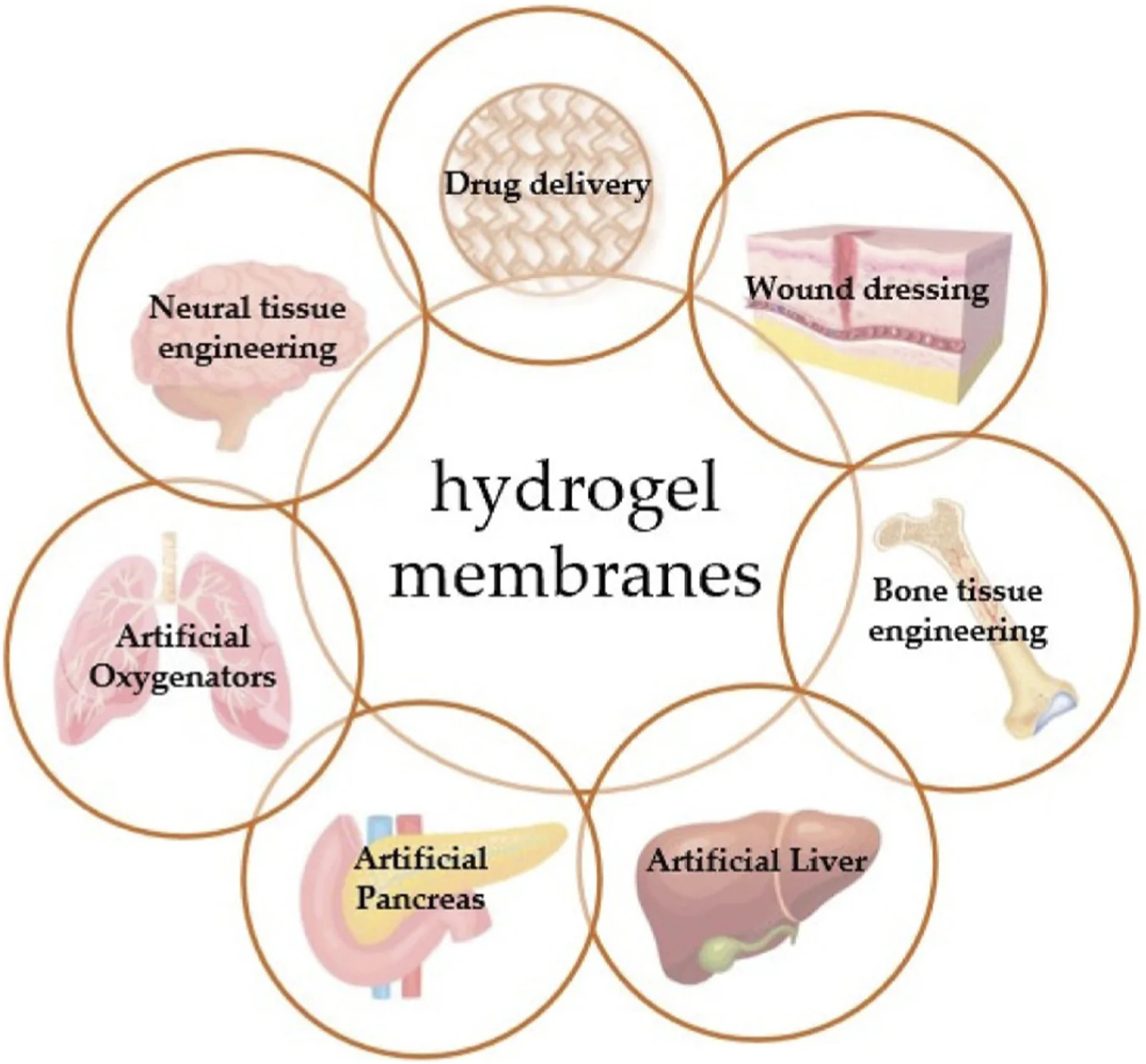
Hydrogel membranes and their application areas in biomedical engineering. Reproduced under license CC-BY-4.0 (Trombino et al., 2023b).

In hydrated polymer networks, the mechanical response is strongly influenced by polymer concentration, crosslinking density, network homogeneity, and the presence of entanglements or secondary physical interactions. Increasing crosslinking density generally increases elastic stiffness and limits swelling but may reduce extensibility and alter the network mesh size, thereby affecting the transport of water, ions, and therapeutic molecules. Network heterogeneity and weakly connected regions may also contribute to localised deformation, stress concentration, and damage under repeated loading. Accordingly, hydrogel membranes should be characterised in their hydrated state, with mechanical testing and permeability measurements considered together when evaluating their suitability for drug delivery, wound care, and soft-tissue interfaces.

### 3.5 Fluoropolymers and functional polymer films

Expanded polytetrafluoroethylene (ePTFE) and polyvinylidene fluoride (PVDF) serve distinct functional purposes. The unique node-fibril microstructure of ePTFE provides a vascular graft that is axially compliant yet radially stiff, boasting excellent hemocompatibility. Additionally, its mechanical anisotropy is intrinsically aligned with the mechanics of arterial walls. Meanwhile, as piezoelectric membrane materials, PVDF and its copolymer PVDF-TrFE are optimally suited to directly convert biomechanical strain into electrical charge. Through *in situ* electrical poling of electrospun PVDF-TrFE nanofiber membranes, β-phase crystallite fractions exceeding 90% and piezoelectric coefficients (d_33_) of up to −57 pC/N are achieved. This allows for self-powered biosensing from physiological movements, such as breathing and cardiac pulsation, with pressure sensitivities around 1.1 V/kPa (Hwang et al., 2015; Khan et al., 2025). Moreover, incorporating reduced graphene oxide (rGO) or MXene into PVDF matrices via electrospinning significantly enhances both piezoelectric output and mechanical stiffness through preferential β-phase nucleation (He et al., 2023; Guo et al., 2024).

### 3.6 High-modulus polymers and conductive composites

Polyimides and parylene-based materials are widely used in flexible and implantable electronics because their combination of mechanical stability, thin-film processability, electrical insulation, and chemical resistance enables integration with microfabricated devices. However, their relatively high modulus compared with soft biological tissues makes geometric design and thickness reduction important for controlling bending stiffness. Conductive polymer composites provide an additional design strategy in which mechanical compliance and electrical functionality are combined within a single membrane. Incorporation of conductive fillers or intrinsically conducting polymers can enable strain sensing, electrophysiological recording, or stimulation. However, repeated deformation may alter conductive pathways through filler separation, crack formation, or changes in percolation networks (Souri et al., 2020; Balakrishnan et al., 2022).

The mechanical performance of Parylene and polyimide films depends not only on their intrinsic material properties but also on film thickness, device geometry, interfacial adhesion, and fabrication conditions. For example, Sohal et al. demonstrated the value of geometry-informed design in Parylene-C intracortical probes: finite-element analysis showed that a sinusoidal shaft accommodated displacement more effectively than a straight shaft while reducing the predicted stress in the probe structure (Sohal et al., 2016). This illustrates how device architecture can compensate for the relatively high elastic modulus of a polymer compared with soft neural tissue. Similarly, Guo et al. developed an ultrathin polyimide-based neural interface and combined mechanical characterisation with computational bending analysis, demonstrating that substrate thickness is an important design variable for reducing bending stiffness (Guo et al., 2022).

Long-term reliability also requires consideration of material ageing and interface integrity. Rubehn and Stieglitz evaluated three commercially available polyimides under prolonged phosphate-buffered saline exposure and accelerated ageing, showing that stability depended on the polymer formulation and exposure conditions (Rubehn and Stieglitz, 2010). More recently, Tintelott et al. investigated polyimide-based neural implant structures and demonstrated that interfacial pretreatment influenced insulation stability during accelerated ageing (Tintelott et al., 2022). Collectively, these findings indicate that thin-film selection should consider tensile and flexural behaviour, thickness-dependent stiffness, adhesion, environmental stability, and device-level reliability rather than relying on nominal elastic modulus alone.

### 3.7 Molecular and microstructural determinants of membrane mechanics

The mechanical performance of polymeric membranes is governed not only by polymer chemistry but also by molecular weight, chain architecture, crystallinity, crosslinking density, phase morphology, and the interactions between constituent phases (Nunes et al., 1982; Liu et al., 2006). These parameters influence chain mobility, intermolecular interactions, load transfer, molecular relaxation, and the development of defects, thereby determining the macroscopic stiffness, strength, toughness, fatigue resistance, dimensional stability, and permeability of the membrane. Their effects are interdependent and vary with polymer chemistry, processing history, temperature, hydration, and loading conditions (Mousavi et al., 2023).

Increasing molecular weight generally increases chain entanglement and can improve tensile strength, ductility, and resistance to crack propagation, particularly when the polymer molecular weight approaches or exceeds the entanglement molecular weight (Karger-Kocsis and Moskala, 2000). However, higher molecular weight may also increase melt viscosity and complicate film formation or pore control during processing. Chain architecture further influences mechanical response: linear, branched, segmented, and network polymers differ in chain mobility, physical entanglement, and the ability to dissipate energy under deformation. In segmented polyurethanes, for example, the relative proportions and organisation of hard and soft segments influence modulus, elasticity, and energy dissipation (Ji et al., 2024; Yi et al., 2026). These relationships are important when designing membranes that must accommodate repeated deformation without excessive permanent strain or premature failure. Crystalline domains can act as physical crosslinks and reinforcing regions within a polymer, generally increasing stiffness, strength, and dimensional stability. However, increased crystallinity may reduce extensibility, depending on the polymer system and crystalline morphology. In biodegradable polyesters, crystallinity also influences water uptake, hydrolytic degradation, and the retention of mechanical integrity during resorption (Nair and Laurencin, 2007). In semicrystalline or phase-separated polymers, the size, distribution, orientation, and connectivity of the constituent phases determine how stresses are transferred through the membrane. Poor interphase adhesion or heterogeneous morphology may promote local stress concentrations and crack initiation, whereas a well-organised morphology can improve load transfer and deformation stability. Consequently, crystallinity and phase morphology should be considered alongside bulk modulus when selecting polymers for load-bearing, flexible, or degradable membrane applications.

In elastomers and hydrogels, crosslinking density controls the mobility of polymer chains between network junctions. Increasing crosslink density commonly increases elastic modulus and dimensional stability while reducing extensibility and equilibrium swelling. In hydrated networks, crosslinking and polymer concentration also influence mesh size and molecular transport, so changes intended to improve mechanical integrity may reduce permeability or alter the release of therapeutic molecules. Conversely, insufficient crosslinking can result in excessive swelling, creep, or loss of structural integrity. The appropriate network structure therefore depends on the balance between load-bearing requirements, reversible deformation, hydration, and transport functionality. Molecular-scale changes become particularly important when translated into thin-film or porous membrane structures. Stiffness depends on both the intrinsic material response and the geometry of the membrane, whereas toughness and fatigue resistance additionally depend on defects, interfaces, morphology, and the mechanisms available for dissipating energy. Permeability is influenced by polymer-solute interactions, free volume, network mesh size, porosity, and the connectivity and tortuosity of transport pathways. These variables may interact: increasing crystallinity or crosslinking can improve dimensional stability but restrict molecular transport, while introducing pores or a second phase can increase permeability but reduce the effective load-bearing area or create stress concentrations. For smart medical devices, the resulting design must therefore balance mechanical durability, deformation stability, transport, and functional performance rather than optimise any single property in isolation.

Accordingly, a structure-property assessment should connect molecular and microstructural parameters to membrane-level measurements under application-relevant conditions. Useful measurements include tensile and flexural properties, fracture or tear resistance, cyclic fatigue, creep, swelling, degradation, and permeability. Where appropriate, these measurements can be complemented by microscopy, thermal analysis, and computational modelling to relate morphology and molecular organisation to the observed response. Such an integrated assessment provides a mechanistic basis for selecting polymer compositions and processing conditions for membranes intended for specific physiological environments.

## 4 Manufacturing and architecture: how processing controls mechanics

The mechanical behaviour of a polymeric membrane is governed not only by its intrinsic polymer properties but also by the architecture generated during fabrication. Processing parameters can control thickness, porosity, fibre orientation, pore morphology, crystallinity, layer adhesion, surface topography, and internal defects, thereby producing substantial differences in stiffness, strength, fatigue resistance, permeability, and interfacial behaviour even when the same polymer is used. Consequently, manufacturing should be considered an integral component of biomechanics-guided design rather than a downstream processing step. The following sections highlight four manufacturing strategies (see Table 3) in which processing parameters can be deliberately used to tailor membrane mechanics and architecture.

**TABLE 3:** Manufacturing strategies, architectural features, and biomechanical consequences of polymeric membranes.

Manufacturing strategy	Architecture controlled	Main mechanical effect	Key trade-off	Representative applications
Electrospinning	Fibre diameter, alignment, porosity	Anisotropy, flexibility, toughness	Porosity vs. strength	Cardiac, vascular, wound
Phase inversion	Pore size, skin layer, porosity	Compaction, creep stability	Permeability vs. mechanical stability	Dialysis, filtration
Additive manufacturing	Geometry, layers, gradients, infill	Spatially tunable mechanics	Resolution vs. strength/layer adhesion	Patient-specific devices
Thin-film deposition	Thickness, density, residual stress	Bending stiffness, barrier behaviour	Conformability vs. durability	Neural/electronic coatings
Surface patterning	Micro/nanotopography	Interfacial mechanics, cell interaction	Pattern fidelity vs. scalability	Biosensors, mechanosensing

### 4.1 Electrospinning

Electrospinning is particularly valuable for producing thin, highly porous polymeric membranes with controllable fibre diameter and orientation. The resulting fibrous architecture can reproduce important structural characteristics of biological tissues, particularly those containing aligned collagen networks. Collector rotation, electric-field conditions, solution properties, and processing rate can be adjusted to control fibre alignment and, consequently, the directional mechanical response of the membrane. A major advantage is the ability to introduce mechanical anisotropy without substantially changing polymer chemistry. Aligned fibres can provide higher stiffness and strength along the preferred direction, making electrospinning relevant to vascular, cardiac, tendon, and other anisotropic tissue interfaces (Bhardwaj and Kundu, 2010; Schoen et al., 2017). However, high porosity can reduce mechanical strength, creating a trade-off between permeability, cellular infiltration, and structural integrity. Your existing manuscript identifies porosities of approximately 60%–90% for electrospun membranes, and highlights approaches such as coaxial spinning, polymer blending, and nanoparticle reinforcement to address this trade-off (Sill and Von Recum, 2008). Thus, electrospinning provides a direct route from processing parameters to biological function via fibre architecture and anisotropic mechanics.

The process-structure-property relationship in electrospinning is determined by the combined effects of solution properties, electric-field conditions, flow rate, tip-to-collector distance, and collector motion. Polymer concentration and solution viscosity influence chain entanglement and fibre formation, whereas solvent volatility and environmental conditions affect solvent evaporation and fibre morphology. Applied voltage, flow rate, and tip-to-collector distance interact to determine jet stretching, fibre diameter, and the occurrence of beads or other morphological defects. Collector speed and geometry influence fibre alignment and, consequently, the directional mechanical response of the resulting membrane. These architectural changes affect not only tensile stiffness and strength but also pore connectivity, permeability, and deformation under repeated loading. For example, increased fibre alignment can improve load-bearing capacity along the preferred direction but may reduce transverse reinforcement, whereas increased porosity can facilitate transport and cellular infiltration while reducing the effective load-bearing area. Therefore, electrospinning parameters should be optimised jointly to achieve the required combination of anisotropy, permeability, mechanical integrity, and fatigue resistance.

### 4.2 Phase inversion

Phase inversion is widely used for porous and asymmetric membranes, particularly in filtration, dialysis, and oxygenation applications. During fabrication, polymer precipitation following solvent-nonsolvent exchange generates a characteristic skin-sublayer structure. Polymer concentration, solvent/nonsolvent composition, additive concentration, and exchange kinetics determine pore size, porosity, skin thickness, and ultimately the mechanical and transport properties of the membrane. From a biomechanics perspective, an important issue is mechanical compaction under sustained transmembrane pressure (TMP). Deformation and collapse of the porous structure can alter permeability and selectivity over time, linking creep behaviour directly to membrane function (Sill and Von Recum, 2008). Reinforcing fillers, asymmetric architectures, and controlled pre-compaction can therefore improve mechanical stability while preserving transport performance. Phase inversion consequently illustrates the strong mechanics-transport coupling that is particularly important for membranes exposed to sustained pressure or fluid flow.

In phase inversion, membrane mechanics are strongly influenced by the kinetics of solvent-nonsolvent exchange and the resulting pore architecture. Polymer concentration, solvent-nonsolvent affinity, bath composition and temperature, additive content, and coagulation conditions can alter the formation of the surface skin, the thickness and connectivity of the porous sublayer, and the development of macrovoids. These features determine the distribution of load-bearing material through the membrane thickness and influence tensile strength, compressive stability, and resistance to pore collapse. A dense surface layer may provide dimensional stability and selective transport, whereas a highly porous sublayer can reduce mechanical resistance under sustained pressure. Under prolonged loading, deformation or compaction of the porous region may change pore size and connectivity, resulting in time-dependent changes in permeability and selectivity. Accordingly, phase-inversion conditions should be selected by balancing the required separation or transport performance with sufficient structural stability under the intended pressure, flow, and operating duration.

### 4.3 Additive manufacturing

Additive manufacturing (AM) enables membrane and thin-film architectures that are difficult to achieve using conventional processing, including patient-specific geometries, spatially varying porosity, graded structures, and multilayer configurations. In biomedical applications, this capability is particularly relevant when the membrane must reproduce complex anatomical geometries or spatially varying mechanical environments. Process parameters such as printing path, layer thickness, infill density, printing orientation, deposition rate, and interlayer bonding can influence the final mechanical response. Consequently, nominal polymer properties may not represent the effective properties of the printed membrane. Anisotropy can arise from the deposition direction, while imperfect interlayer bonding can become a critical fatigue initiation site. Three-dimensional printing can therefore be used to engineer spatially heterogeneous mechanical properties, including gradient stiffness and porosity. However, reproducibility, resolution, surface quality, post-processing, and long-term layer-adhesion fatigue remain important considerations for biomedical translation (Uhljar and Ambrus, 2023).

The influence of additive-manufacturing parameters on membrane mechanics is mediated by the geometry and integrity of the deposited structure. Layer thickness, raster or printing orientation, infill density, deposition rate, and the thermal history between successive layers determine the continuity of deposited paths, the size and distribution of voids, and the degree of interlayer bonding. A higher infill density may increase effective stiffness and strength by increasing the load-bearing fraction, but it can also reduce permeability and flexibility. Similarly, aligned deposition paths can produce direction-dependent mechanical properties, while weak interlayer interfaces or incomplete fusion can promote delamination and fatigue damage. Graded porosity and spatially varying architectures offer opportunities to distribute deformation or stiffness according to application-specific requirements; however, the intended gradients must be verified in the manufactured structure rather than inferred solely from the digital design. Mechanical testing in relevant orientations, together with assessment of interlayer integrity and transport properties, is therefore essential when translating printed membrane architectures into reliable biomedical devices.

### 4.4 Thin-film deposition and surface patterning

For ultrathin biomedical membranes, mechanical behaviour is strongly influenced by film thickness, residual stress, substrate constraint, and surface properties. Physical vapour deposition (PVD) and chemical vapour deposition (CVD) can generate dense, conformal films ranging from nanometres to micrometres in thickness and are particularly useful for protective, insulating, and haemocompatible coatings (Khlyustova et al., 2020). Because bending stiffness decreases strongly with decreasing thickness, ultrathin films can achieve high conformability even when their bulk polymer or coating material is relatively stiff. However, residual stresses and poor adhesion between the film and the substrate can lead to cracking, buckling, or delamination under cyclic deformation. Techniques such as nanoimprint lithography, microtexturing, and related lithographic approaches can modify surface roughness, contact area, wettability, and local mechanical interactions with cells. Submicrometre topographies can also influence cellular mechanotransduction and adhesion, providing a route for coupling membrane mechanics with biological response. Thus, thin-film deposition and patterning extend biomechanics-guided design from bulk mechanical properties to interfacial and nanoscale mechanics.

For deposited thin films, mechanical behaviour depends on the interaction between film thickness, deposition conditions, substrate properties, residual stress, and interfacial adhesion. Deposition rate, substrate temperature, plasma or surface-treatment conditions, and subsequent processing can influence film density, microstructure, defect formation, and adhesion. These factors determine how the film accommodates bending and in-plane deformation and whether stresses are relieved through elastic deformation or localised damage. Reducing film thickness can substantially decrease bending stiffness, improving conformity to curved or moving tissues, although defects and interfacial weaknesses may become increasingly important in ultrathin structures. Surface patterning can further modify the effective deformation response by introducing geometric features that accommodate strain or redistribute local stresses. However, sharp features, defects, and poorly bonded regions may act as stress concentrators and promote crack initiation or delamination under cyclic loading. Consequently, deposited films should be evaluated in their final substrate-supported configuration, with attention to bending behaviour, residual stress, adhesion, and cyclic reliability rather than relying solely on the bulk properties of the deposited polymer.

## 5 Interfacial and multilayer mechanics

The mechanical performance of polymeric membranes is governed not only by the properties of individual materials but also by how different layers, phases, and functional components interact mechanically. In multilayer membranes, a mismatch in stiffness, swelling, deformation, or thermal expansion can concentrate stresses at interfaces and promote delamination. Similarly, introducing spatial variations in composition or structure can create mechanical gradients that improve load transfer and tissue integration. These mechanical effects are also coupled with mass transport, as deformation can alter porosity, free volume, diffusion pathways, and permeability. Thus, interfacial integrity and mechanics-transport coupling should be considered together when designing multifunctional membranes. Table 4 shows the interfacial and architectural strategies for controlling the mechanical and transport behaviour of polymeric membranes.

**TABLE 4:** Interfacial and architectural strategies for controlling the mechanical and transport behaviour of polymeric membranes.

Strategy	Structural feature	Mechanical consequence	Transport consequence	Representative applications
Surface functionalization	Covalent/chemical bonding	Improved adhesion, reduced delamination	Can modify surface permeability/wettability	Implant coatings, sensors
Transition/graded layers	Gradual modulus variation	Reduced interfacial stress concentration	Controlled diffusion across layers	Multilayer implants
Fibre alignment	Directional architecture	Anisotropic stiffness and strength	Direction-dependent permeability	Vascular/cardiac membranes
Porosity gradients	Spatially varying pore structure	Local control of compliance	Tunable permeability	Filtration, tissue interfaces
Multilayer architecture	Distinct functional layers	Load sharing and improved durability	Independent control of barrier/transport functions	Drug delivery, wound dressings
Crosslink-density gradients	Spatially varying polymer network	Tunable stiffness and swelling	Controlled diffusivity	Hydrogels, drug delivery

### 5.1 Interfacial adhesion and delamination

Multilayer membranes frequently combine materials with substantially different mechanical properties, such as a stiff substrate, a compliant polymer layer, a conductive coating, or a bioactive surface. Although these combinations provide complementary functionality, differences in modulus, strain, swelling, and thermal expansion can generate stress concentrations at interfaces. Under repeated physiological loading, these stresses may initiate interfacial cracking and progressive delamination, even when the individual layers remain mechanically intact. Interfacial performance is therefore better described by adhesion strength and interfacial fracture toughness than by the bulk properties of either material alone. Mode-I and mode-II interfacial fracture energies, commonly represented by $G_{IC}$ and $G_{IIC}$ , provide useful measures of resistance to crack opening and shear-driven separation, respectively (Hutchinson and Suo, 1991). The coatings on drug-eluting stents, which consist of 5–15 µm polyurethane (PU) or poly (butyl methacrylate) (PBMA) films applied to metallic struts, expand radially by 20%–40% when the stent is deployed using a balloon. After deployment, these coatings undergo pulsatile bending at approximately 40 million cycles per year (James and Sire, 2010; Ono et al., 2011). In clinical cases, late stent thrombosis has been linked to viscoelastic mismatch and residual thermal stresses, which can lead to delamination. Therefore, for flexible biomedical devices, the interface must withstand not only peak loading but also millions of relatively small deformation cycles.

Several strategies can improve interfacial stability. Covalent coupling agents, surface functionalization, plasma treatment, micro- and nanotexturing, and intermediate adhesion layers can enhance chemical or mechanical bonding between dissimilar materials. A particularly useful approach is to introduce a graded transition layer, which reduces the abrupt modulus mismatch between a rigid substrate and a compliant polymer. In nanocomposite membranes, functionalization of fillers can similarly improve polymer-filler adhesion and reduce filler pull-out under cyclic deformation (Liu et al., 2023). Importantly, interfacial design should account for the wet and physiologically conditioned state, as hydration, protein adsorption, swelling, and degradation can alter interfacial strength over time. Consequently, adhesion should be evaluated under representative cyclic, thermal, chemical, and aqueous conditions rather than only through initial dry-state measurements.

### 5.2 Mechanical gradients and heterogeneous architectures

Biological tissues rarely exhibit uniform mechanical properties. Instead, they contain gradients in stiffness, porosity, composition, and anisotropy across length scales. Reproducing such heterogeneity in polymeric membranes can improve mechanical compatibility and reduce abrupt transitions between a device and surrounding tissue (Asgharzadeh Shirazi et al., 2017; Pattnaik et al., 2023). Mechanical gradients can be generated through changes in polymer composition, crosslink density, fibre orientation, porosity, layer thickness, or incorporation of reinforcing phases. For example, a membrane can be designed with a compliant tissue-facing layer and a mechanically stronger supporting layer, while an intermediate region provides gradual load transfer. Similarly, spatial control of fibre alignment can produce direction-dependent stiffness that more closely resembles anisotropic tissues such as blood vessels, myocardium, and tendons (Oroujzadeh et al., 2024).

Compared with conventional homogeneous films, heterogeneous architectures offer an additional design variable: the location of mechanical properties within the membrane. This can be particularly important for devices subjected simultaneously to bending, tension, fluid pressure, and interfacial shear. However, gradients must be sufficiently continuous or mechanically stable to avoid creating new stress concentrations at internal transitions. The design objective is therefore not simply to match a single tissue modulus, but to reproduce the spatial distribution of mechanical response relevant to the application. Combining graded mechanics with controlled porosity and surface functionality may consequently provide a more effective strategy for tissue integration and long-term mechanical stability.

### 5.3 Mechanics-transport coupling

For membranes used in drug delivery, dialysis, filtration, wound management, and oxygenation, mechanical deformation can directly modify transport behaviour. Conversely, fluid uptake, swelling, solute diffusion, and degradation can alter the membrane’s mechanical properties. This creates a bidirectional mechanics-transport coupling that must be considered during design. In dense polymer films, tensile deformation can increase intermolecular spacing and free volume, potentially increasing molecular diffusivity (Merkel et al., 2000; Di et al., 2015). In porous membranes, mechanical compression can reduce pore size and effective porosity, thereby decreasing permeability. This effect is particularly relevant to hollow-fibre and pressure-driven systems, where sustained transmembrane pressure can produce creep and compaction. The resulting reduction in pore dimensions can progressively alter permeability and selectivity during operation (Li et al., 2020).

Swelling provides another important coupling mechanism. Water or physiological fluid uptake can increase membrane dimensions and decrease effective polymer-chain interactions, producing changes in stiffness, viscoelasticity, and transport pathways (Caccavo et al., 2018; Richbourg and Peppas, 2020). Similarly, degradation may progressively reduce molecular weight or network integrity while simultaneously increasing porosity or creating new diffusion pathways. These changes mean that transport properties measured immediately after fabrication may not represent long-term behaviour under physiological loading (Umemori and Little, 2025). A biomechanics-guided membrane design should therefore evaluate permeability, diffusivity, and mechanical properties under coupled conditions rather than independently. Useful experimental approaches include simultaneous mechanical-permeation testing, cyclic pressure loading, wet-state tensile testing, and time-dependent measurements during swelling or degradation. This integrated approach is particularly important for membranes in which mechanical deformation is itself used to regulate transport, such as mechanically triggered drug-delivery systems and electroactive or piezoelectric membranes (Zhang et al., 2016; Ma et al., 2022).

Experimental and simulation studies demonstrate why material properties should be evaluated in the context of the complete device. In flexible neural probes, Singh et al. combined finite-element modelling with experimental insertion tests to investigate the influence of probe dimensions and sacrificial coating thickness on buckling and successful insertion. Their findings show that flexibility alone is insufficient: a device must retain adequate insertion stability while minimising mechanical disturbance to surrounding tissue (Singh et al., 2016). In polyimide-based neural implants, long-term immersion studies and accelerated electrical testing further demonstrate that environmental exposure and interfacial integrity can affect reliability even when the initial material properties are suitable (Rubehn and Stieglitz, 2010; Tintelott et al., 2022). These findings have broader implications for polymeric membranes used in smart medical devices. Mechanical characterisation should, wherever relevant, be complemented by application-specific tests that reproduce the dominant loading modes, fluid environment, and duration of use. Depending on the device, these may include cyclic bending or tensile loading, wet-state testing, adhesion assessment, accelerated ageing, and coupled mechanical-transport measurements. Computational models can then be used to examine stress concentrations, deformation, and design variations that are difficult to isolate experimentally. However, predictions should be interpreted alongside experimental validation and the assumptions used to represent material behaviour and physiological loading.

## 6 Smart and stimuli-responsive membranes

Smart polymeric membranes extend conventional passive devices by converting physiological or environmental stimuli into mechanical, electrical, chemical, or therapeutic responses. For biomedical applications, the most relevant stimuli include mechanical deformation, temperature, pH, magnetic fields, and electrical signals (see Figure 4). Their integration with thin-film and membrane architectures enables simultaneous sensing, actuation, and controlled delivery while maintaining flexibility and tissue compatibility (Mohammadzadeh et al., 2026). From a biomechanics perspective, the key design principle is that the stimulus must be efficiently coupled to the membrane. Consequently, mechanical sensitivity, response stability, reversibility, and fatigue resistance are as important as the magnitude of the functional output (Stuart et al., 2010).

**FIGURE 4:**
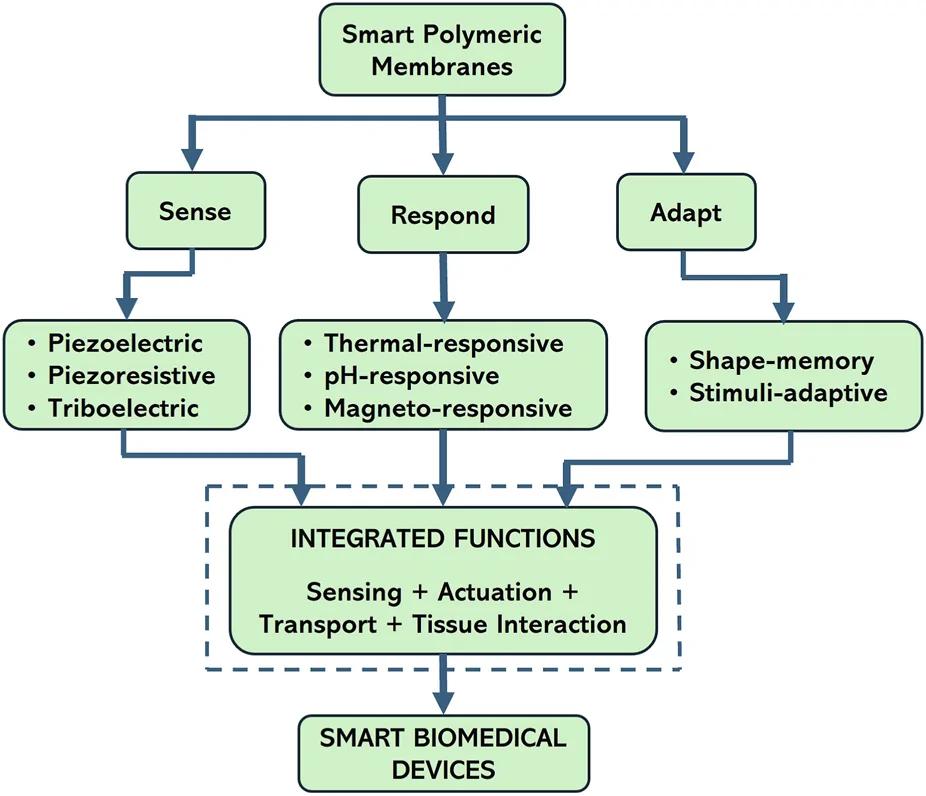
Functional classification of smart polymeric membranes.

### 6.1 Mechanically active sensing

Mechanical sensing membranes convert deformation generated by physiological activities into measurable electrical or optical signals. Sources of deformation include stretching, compression, bending, shear, and cyclic pressure associated with cardiac contraction, respiration, joint movement, vascular pulsation, and skin deformation. Polymeric membranes are particularly suitable for these applications because their low bending stiffness and high extensibility allow them to conform to curved and dynamically moving biological surfaces (Shen et al., 2022). Conductive polymers, elastomer-nanomaterial composites, and hydrogel-based networks can be engineered to respond to mechanical deformation by changing electrical resistance, capacitance, impedance, or other measurable properties. Materials such as PEDOT:PSS, carbon nanotubes, graphene, MXenes, and conductive hydrogels can be incorporated into elastomeric or hydrogel matrices to produce flexible strain and pressure sensors. Fibre alignment, crack-like conductive pathways, porous structures, and multilayer configurations can amplify local deformation, thereby increasing sensitivity. However, highly sensitive architectures may also exhibit greater hysteresis, nonlinear response, or mechanical degradation under repeated loading. Therefore, sensor design should consider not only sensitivity but also gauge factor, detection range, hysteresis, response time, repeatability, and fatigue life (Sharma et al., 2022; Yadav et al., 2023).

### 6.2 Piezoelectric and triboelectric membranes

Piezoelectric membranes provide a direct route for converting physiological mechanical deformation into electrical signals. Poly (vinylidene fluoride) (PVDF) and its copolymer PVDF-TrFE are widely investigated because their electroactive phases generate electrical charge in response to stretching, compression, and bending. Electrospinning, mechanical stretching, electrical poling, and incorporation of functional nanomaterials can modify polymer crystallinity, phase composition, fibre orientation, and electromechanical response. Reported $d_{33}$ values for electrospun PVDF-TrFE and MXene-containing composites are approximately −33 to −57 pC/N, demonstrating their potential for converting relatively small physiological deformations into measurable electrical outputs (Khan et al., 2025; Kim et al., 2025). The compatibility between the mechanical operating range of the membrane and physiological loading is particularly important. Cardiac and respiratory activities occur at characteristic frequencies of approximately 1 Hz and 0.3 Hz, respectively, providing representative low-frequency mechanical inputs for wearable and implantable monitoring. Triboelectric membranes use a different transduction mechanism based on contact electrification and electrostatic induction between dissimilar materials. PDMS/polyimide-based triboelectric nanogenerators, for example, can convert repeated contact-separation, sliding, or deformation into electrical signals. Their output is strongly influenced by surface chemistry, effective contact area, micro/nanotopography, deformation amplitude, and interfacial mechanics (Kim et al., 2021; Delgado-Alvarado et al., 2022). Surface patterning can increase the effective contact area and enhance electrical output, while the compliant nature of polymeric substrates enables conformal integration with biological surfaces. Although piezoelectric and triboelectric membranes use different physical mechanisms, both depend fundamentally on efficient mechanical-to-electrical coupling. Therefore, membrane stiffness, thickness, fibre or surface architecture, deformation mode, interfacial adhesion, and fatigue resistance should be optimized together with electrical output. This biomechanics-based approach is particularly important for implantable devices, where millions of low-amplitude loading cycles may occur during service (Kim et al., 2021).

### 6.3 Thermo-, pH- and magneto-responsive systems

Stimuli-responsive polymers can undergo changes in swelling, conformation, permeability, or mechanical properties in response to changes in their surrounding environment. These systems provide routes toward membranes that respond autonomously to physiological conditions. Thermoresponsive polymers can undergo reversible phase transitions at specific temperature ranges. Poly (N-isopropylacrylamide) (PNIPAM), for example, exhibits a lower critical solution temperature near physiological temperature, making it attractive for thermally controlled swelling, permeability, and drug release. The transition temperature and mechanical response can be adjusted through copolymer composition, crosslink density, and network architecture. pH-responsive membranes exploit ionizable functional groups whose degree of protonation changes with environmental pH. Polymers containing carboxyl, amine, or other ionizable groups can consequently undergo changes in swelling and permeability. This behaviour is particularly relevant to drug delivery and wound-management systems, where local pH can vary during inflammation, infection, and tissue healing. Magneto-responsive membranes enable non-contact actuation by incorporating magnetic nanoparticles into compliant polymer matrices. Fe_3_O_4_ superparamagnetic iron oxide nanoparticles (SPIONs) embedded in PDMS or polyurethane can undergo magnetically induced deformation in an external magnetic field (Zhang et al., 2020; Elsayed and Teow, 2025). Such magnetoelastic composites have been investigated for remotely controlled drug-delivery systems, mechanical stimulation, and regenerative medicine applications. Their performance depends on nanoparticle concentration, spatial distribution, magnetic-field characteristics, polymer stiffness, and particle-matrix adhesion. Repeated magnetic actuation can generate local interfacial stresses, making mechanical durability an important consideration. Overall, the design of thermo-, pH-, and magneto-responsive membranes requires a balance between stimulus sensitivity and mechanical stability. Swelling, repeated stimulus cycling, particle-matrix interactions, dimensional stability, and long-term reversibility should be evaluated alongside the magnitude and kinetics of the functional response (Wandera et al., 2010; Thévenot et al., 2013).

### 6.4 Mechanically coupled drug delivery

The integration of drug delivery with mechanically responsive membranes provides an opportunity to convert physiological deformation into on-demand therapeutic release. In these systems, stretching, compression, bending, fluid pressure, or other mechanical stimuli can alter membrane permeability, pore dimensions, polymer free volume, or the integrity of a drug-containing reservoir. One approach uses mechanically deformable polymer networks in which strain modifies free volume or diffusion pathways, thereby changing the transport rate of encapsulated molecules. Another uses mechanically triggered rupture, deformation of micro-reservoirs, or pressure-sensitive layers to release a therapeutic payload (Wang et al., 2017). Piezoelectric and electroactive membranes provide an additional route in which mechanical deformation generates an electrical signal that can subsequently control release. The principal advantage of mechanically coupled delivery is that drug release can be linked to the physiological event itself. For example, cyclic deformation associated with movement or tissue loading could regulate release in response to local mechanical demand. However, achieving predictable dose control remains challenging because physiological loading is variable and often heterogeneous. This requires characterisation under realistic cyclic loading rather than static mechanical testing alone. Important parameters include release kinetics, loading amplitude and frequency, fatigue behaviour, swelling, diffusion, drug-polymer interactions, and long-term structural stability (Zhang et al., 2016; Shi et al., 2024).

## 7 Reliability under physiological conditions

The long-term performance of polymeric membranes depends not only on their initial mechanical properties but also on how those properties evolve under repeated loading, sustained deformation, physiological fluids, sterilisation, and biological interactions. Unlike conventional engineering films, biomedical membranes operate in environments where mechanical, chemical, and biological processes occur simultaneously. Cyclic deformation can initiate fatigue damage; sustained loading can produce creep or stress relaxation; and hydration, hydrolysis, oxidation, protein adsorption, and mineral deposition can progressively alter the material’s structure (Lyu and Untereker, 2009; Rondinella and Marin, 2026). Therefore, reliability assessment should evaluate the membrane in a physiologically relevant state rather than relying solely on initial mechanical measurements.

### 7.1 Fatigue and cyclic loading

Many biomedical membranes experience millions of loading cycles during their service life. Cardiac and vascular devices undergo repetitive pulsatile deformation, whereas neural interfaces, wound dressings, wearable sensors, and joint-related devices experience repeated stretching, bending, compression, or shear. Consequently, fatigue resistance is often more relevant to long-term performance than the initial tensile strength. Fatigue damage commonly initiates at material defects, pores, notches, fibre intersections, filler agglomerates, or interfaces between mechanically dissimilar layers. Under repeated loading, these local stress concentrations can promote microcrack formation, followed by crack growth and eventual membrane failure. Fatigue behaviour can be evaluated through cyclic tensile, bending, pressure, or biaxial testing under physiologically representative loading conditions. Crack-growth approaches can additionally describe the relationship between cyclic driving force and crack propagation, for example, through Paris-type relationships (Equation 1) (Anderson and Anderson, 2005):

$$\frac{da}{dN}=C{\left(\Delta K\right)}^{m}$$ (1)

where a is crack length, N is the number of cycles, ΔK is the cyclic stress-intensity range, and C and m are material-dependent parameters. For thin and highly deformable membranes, however, large-strain viscoelasticity and nonlinear fracture behaviour may require approaches beyond conventional linear-elastic fracture mechanics. Importantly, fatigue testing should account for hydration, temperature, frequency, mean strain, stress amplitude, and physiological fluid exposure, because these factors can substantially modify fatigue life. A membrane that performs adequately under dry laboratory conditions may exhibit markedly different behaviour after prolonged exposure to an aqueous biological environment.

### 7.2 Creep and stress relaxation

Polymeric membranes exhibit time-dependent mechanical behaviour because polymer chains can rearrange under sustained loading. Two important manifestations are creep, in which deformation increases under approximately constant stress, and stress relaxation, in which stress decreases under approximately constant strain. These processes are particularly important for membranes subjected to continuous pressure, fixation, pre-stretching, or sustained tissue contact (Caccavo et al., 2018). In pressure-driven systems, for example, sustained transmembrane pressure can compact porous structures, reducing pore dimensions and permeability. In implantable or tissue-contacting membranes, creep may produce dimensional changes that alter load distribution or interfacial contact. Similarly, stress relaxation can reduce the force transmitted by a membrane that is initially pre-strained, potentially changing the mechanical stimulus delivered to surrounding tissue (Zhang et al., 2026). The time-dependent response can be represented using viscoelastic models such as generalised Maxwell or Kelvin-Voigt frameworks, although more complex constitutive descriptions may be required for large deformation, swelling, or biological environments. Experimentally, creep and relaxation should be characterised over timescales relevant to the intended application rather than only over short laboratory intervals. The influence of temperature, hydration, crosslink density, molecular weight, crystallinity, and physical aging should also be considered. In hydrogels and highly hydrated polymers, fluid redistribution and network rearrangement can further couple mechanical relaxation with swelling and transport (Handge, 2017; Fan et al., 2025). Thus, long-term mechanical stability should be evaluated together with changes in membrane permeability and geometry.

### 7.3 Degradation and sterilisation

Biodegradation and environmental degradation can be either intentional or undesirable, depending on the application. Biodegradable membranes based on polymers such as PLA, PLGA, and PCL are designed to undergo hydrolytic degradation, whereas permanent membranes must retain structural and functional stability over their intended service life. Degradation can change molecular weight, crystallinity, crosslink density, surface chemistry, mass, and mechanical properties. A membrane may retain its apparent shape while experiencing substantial reductions in molecular weight and fatigue resistance. For biodegradable systems, the timing of mechanical loss should therefore be considered relative to the rate of tissue regeneration (Woodruff and Hutmacher, 2010; Saidin et al., 2026). Premature loss of load-bearing capacity can create a mechanical mismatch between the degrading membrane and the developing tissue. Common approaches such as gamma irradiation, electron-beam irradiation, ethylene oxide, steam/autoclave treatment, and other low-temperature sterilisation methods can affect polymer molecular structure, crystallinity, crosslinking, residual stresses, and surface chemistry. Radiation may induce chain scission or crosslinking, depending on the polymer chemistry and processing conditions, while elevated temperatures and moisture can accelerate hydrolytic changes in susceptible polymers. Accordingly, mechanical characterisation should ideally be performed before and after sterilisation, followed by evaluation after relevant storage and physiological conditioning (Delgado et al., 2014; Pérez Davila et al., 2021). This is particularly important for thin membranes because relatively small changes in molecular structure or thickness can influence flexibility, adhesion, permeability, and fatigue performance.

### 7.4 Calcification, fouling and biological responses

Long-term exposure to biological fluids introduces additional failure mechanisms that cannot be predicted from mechanical testing alone. Calcification, protein adsorption, cellular adhesion, thrombosis, biofouling, and inflammatory responses can alter the surface and mechanical behaviour of polymeric membranes. Calcification is particularly relevant to blood-contacting and cardiovascular devices. Mineral deposition can increase local stiffness and reduce membrane flexibility, producing stress concentrations at the interface between mineralised and non-mineralised regions. Continued deposition may therefore promote crack initiation or alter the deformation pattern of the membrane (Thubrikar et al., 1983; Levy et al., 1986). Reported approaches for biomechanical anti-calcification include pre-treatment with amino-oleic acid (AOA), enhancements in polycarbonate-urethane (PCU) chemistry compared to traditional polyether-urethane, and the application of PEG brush layers for surface passivation (Gunatillake et al., 2003; Papathanasiou and Demadis, 2015). Biologically mediated degradation occurs through oxidative attacks by reactive oxygen species (ROS) generated by macrophages, enzymatic hydrolysis, and microbial colonisation, all of which work together with mechanical degradation. Utilising PCU chemistry instead of polyether-urethane addresses concerns related to metal-ion oxidation (MIO) susceptibility while preserving key mechanical properties (Gunatillake et al., 2003). Mechanical qualification testing protocols should consider the effects of sterilisation methods, such as gamma irradiation, which can cause chain scission in polydimethylsiloxane (PDMS) and poly (lactic-co-glycolic acid) (PLGA), and ethylene oxide (EtO) exposure, which can plasticise polymers (Münker et al., 2018).

Biological responses should therefore be considered as part of the mechanical design problem, rather than treated solely as a biocompatibility assessment. Surface chemistry, roughness, stiffness, topography, and mechanical deformation can influence cellular behaviour and protein interactions, while biological deposition can subsequently modify the membrane’s mechanical properties (Shen et al., 2022; Wu et al., 2025). Long-term evaluation should consequently combine mechanical testing with appropriate biological and physicochemical assessments, including protein adsorption, cellular response, mineral deposition, surface morphology, permeability, and changes in mechanical properties. Such coupled testing is essential for establishing whether a membrane can maintain its intended mechanical and functional performance throughout its service period. Table 5 summarises the physiological degradation mechanisms and their implications for polymeric membrane reliability.

**TABLE 5:** Physiological degradation mechanisms and their implications for polymeric membrane reliability.

Reliability challenge	Primary mechanism	Mechanical consequence	Functional consequence	Relevant evaluation
Fatigue	Repeated cyclic loading	Crack initiation and propagation	Loss of structural integrity	Cyclic/fatigue testing
Creep	Sustained loading	Progressive deformation	Geometry and permeability changes	Long-duration creep testing
Stress relaxation	Polymer-chain rearrangement	Reduction in sustained stress	Altered load transfer/actuation	Stress-relaxation testing
Hydrolytic/oxidative degradation	Chemical chain scission or network change	Softening, embrittlement, loss of strength	Altered lifetime and transport	Accelerated/physiological ageing
Sterilisation	Thermal, chemical, or radiation effects	Changes in stiffness, toughness, adhesion	Changed device performance	Pre/post-sterilisation testing
Calcification	Mineral deposition	Local stiffening and stress concentration	Reduced flexibility and fatigue life	Mineralisation and mechanical testing
Protein fouling	Surface adsorption	Altered interfacial mechanics	Changes in transport and biointerface	Protein adsorption/surface analysis
Cellular/inflammatory response	Cell adhesion and biological interaction	Surface and structural changes	Altered biocompatibility and function	*In vitro*/*in-vivo* biological assessment

## 8 Application-based case studies

The preceding sections established a biomechanics-first framework in which physiological loading, polymer chemistry, fabrication architecture, interfacial mechanics, stimulus responsiveness, and long-term reliability are considered as interconnected design variables. The practical value of this framework becomes clearer when applied to different biomedical environments. The following case studies illustrate how the relevant mechanical requirements change across cardiovascular, neural, haemodialysis, and wound-healing applications, and how polymeric membranes and thin films can be engineered accordingly (Wang C. et al., 2024; Yan et al., 2024).

### 8.1 Cardiovascular membranes and valve leaflets

Cardiovascular membranes operate under some of the most demanding combinations of cyclic mechanical loading, fluid pressure, shear stress, and biological exposure. Transcatheter aortic valve replacement (TAVR) leaflets, for example, must repeatedly open and close under pulsatile transvalvular pressure while maintaining sufficient compliance for physiological flow and resistance to fatigue, thrombosis, and calcification. Representative design requirements include peak systolic transvalvular pressures of approximately 100 mmHg, circumferential strains of ∼10%, radial strains of ∼25%, and hundreds of millions of loading cycles over the intended service period (Dasi et al., 2009). Polyurethanes, particularly polycarbonate urethanes (PCUs), have received considerable attention as alternatives to conventional biological or synthetic leaflet materials because their segmented polymer architecture allows the stiffness, elasticity, and toughness to be tailored over a broad range. Fibre-reinforced PCU membranes can further show anisotropic biaxial elastic behaviour mimicking the mechanics of the native aortic valve cusp through controlled fibre orientation (Pilecco et al., 2021). Electrospun PCU meshes combined with hydrogel surface layers have demonstrated anisotropic biaxial behaviour, with reported circumferential and radial moduli of approximately 1.5 and 0.4 MPa, respectively, providing a route toward reproducing the directional mechanical response of native aortic valve tissue (Pilecco et al., 2021; Ghanbari et al., 2009). Accelerated fatigue testing was conducted to 400 million cycles at 700 bpm, and subsequent post-test histological analysis confirmed the sub-critical microstructural integrity and reduced potential for calcification (Claiborne et al., 2013).

Computational modelling provides an important component of the design process. Finite-element analysis can be used to evaluate leaflet deformation, stress distribution, coaptation, and fatigue-related stress concentrations, while fluid-structure interaction approaches can examine the relationship between leaflet mechanics and transvalvular flow. Such models can incorporate anisotropic constitutive descriptions, including Holzapfel-Gasser-Ogden-type formulations, to represent fibre-reinforced tissue behaviour (Wang et al., 2024b). Long-term reliability remains a major challenge, where repeated flexure can initiate microstructural damage, while calcification and thrombotic deposition can progressively alter leaflet stiffness and local stress distributions. Consequently, accelerated fatigue testing and subsequent structural or histological assessment are important components of the evaluation of polymeric valves. The cardiovascular example, therefore, demonstrates the need to simultaneously optimise anisotropy, compliance, fatigue resistance, haemocompatibility, and resistance to biological surface modification, rather than targeting a single mechanical property (Bezuidenhout et al., 2015).

### 8.2 Neural interfaces

Brain tissue is extremely compliant, with reported moduli in the low-kPa range, whereas conventional microelectronic materials such as silicon have moduli on the order of hundreds of gigapascals (E ∼200 GPa). This substantial mismatch can produce mechanical stress concentrations and micromotion at the tissue-device interface, contributing to chronic tissue responses and progressive deterioration of recording performance (Subbaroyan et al., 2005; Lacour et al., 2016). Polymeric thin films provide a strategy for reducing this mechanical mismatch. Polyimide, Parylene-C, PDMS, and hydrogel-based materials can provide substantially lower bending stiffness than conventional rigid substrates while maintaining the structural and electrical functions required for neural recording and stimulation (Liu et al., 2023). Importantly, reducing thickness can decrease bending rigidity dramatically because bending stiffness scales strongly with film thickness. Consequently, an ultrathin polymer substrate can be mechanically compliant even when its intrinsic elastic modulus remains higher than that of brain tissue. Flexible polymeric probe substrates, injectable mesh electronics, and polymer-supported nanowire architectures distribute deformation over larger areas and reduce localised mechanical constraint. For example, GelMA- or PNIPAM-based coatings can provide tissue-like hydrated mechanical environments while simultaneously serving as interfaces for biological integration (Shen et al., 2022). The performance of a neural membrane is determined not simply by the modulus of the bulk polymer but by the combined effects of substrate thickness, electrode geometry, encapsulation, adhesion, hydration, and tissue micromotion. Long-term reliability, therefore, requires simultaneous consideration of mechanical compliance, electrical stability, moisture resistance, adhesion, and biological response. The design objective is consequently better described as minimising mechanical disturbance to the neural tissue while maintaining sufficient structural integrity and signal quality.

### 8.3 Haemodialysis membranes

Polysulfone (PSU)/polyvinylpyrrolidone (PVP) blends and polyethersulfone (PES)-based membranes are widely used because their polymer chemistry and phase-inversion processing permit control over pore structure and surface properties. The asymmetric skin-sublayer architecture produced during phase inversion is particularly important because it allows a thin, selective layer to be supported by a more porous structure. However, the same porous architecture introduces mechanical susceptibility to compaction and creep under sustained TMP, which can progressively reduce pore dimensions and alter permeability (Bezuidenhout et al., 2015). This provides a direct example of the mechanics-transport coupling discussed in Section 5. A mechanically stable membrane must retain its pore structure while maintaining adequate hydraulic permeability and solute selectivity. Surface modification, including heparinisation or zwitterionic functionalization, can additionally reduce protein adsorption and biofouling. Representative haemodialysis conditions involve blood-side shear stresses of approximately 5–50 dyn/cm^2^, together with cyclic and sustained TMP loading. Thus, performance cannot be evaluated solely through initial permeability measurements. Long-duration testing should simultaneously assess mechanical compaction, permeability loss, protein fouling, and solute clearance. The haemodialysis membrane, therefore, provides a particularly clear example of how microstructure, viscoelasticity, transport, and biological interactions must be optimised together rather than independently (Stamatialis et al., 2008; Westphalen et al., 2020).

### 8.4 Smart wound dressings

Wound dressings provide an important example in which a polymeric membrane must simultaneously accommodate large tissue deformation, moisture transport, mechanical protection, sensing, and therapeutic delivery. Unlike rigid implantable devices, a wound dressing must conform to a continuously changing surface and tolerate repeated stretching generated by normal skin movement. Skin can experience strains of approximately 20%–50%, depending on anatomical location and activity, making compliance and extensibility important design requirements. Multilayer and composite polymeric systems provide opportunities to combine these functions. MXene-cellulose/polyurethane composites, for example, can provide mechanical compliance together with electrical conductivity for strain or biochemical sensing, while hydrogel-based layers can provide hydration and controlled transport. They also show interesting properties due to their complex bonding and electronic structures (see Figure 5) (Zarepour et al., 2024). Conductive hydrogel components can be integrated with pH-responsive or drug-loaded layers to create dressings that simultaneously monitor wound conditions and deliver therapeutic agents (Fang et al., 2025; Wang et al., 2025).

**FIGURE 5:**
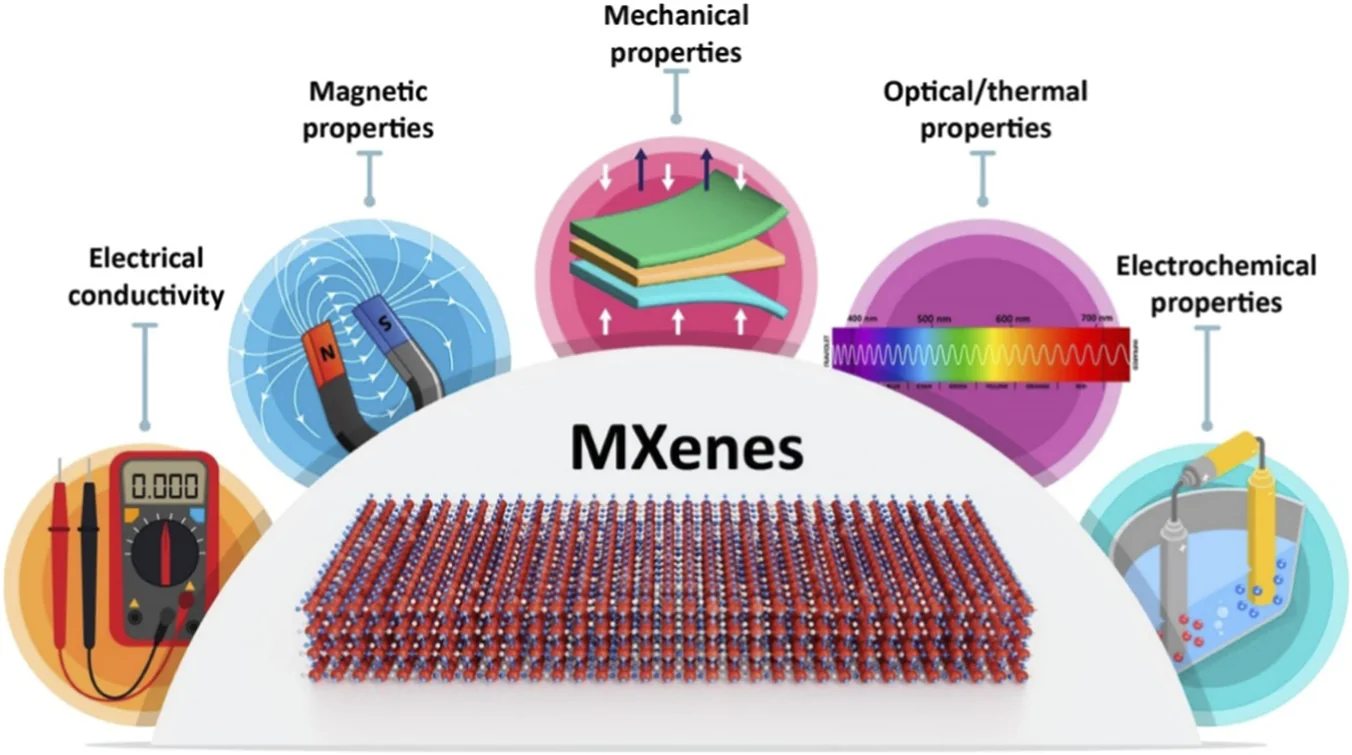
MXenes and their important properties. Reproduced under license CC-BY-4.0 (Zarepour et al., 2024).

From a biomechanics perspective, the key design challenge is to avoid treating the sensing or drug-delivery component as mechanically independent from the dressing. A mechanically mismatched layer can restrict skin movement, delaminate during repeated deformation, or alter wound-device contact. Smart wound dressings also demonstrate the interaction between mechanics and biological state, as the changes in local pH, hydration, exudate, inflammation, and infection can alter the properties of responsive polymer networks and can subsequently trigger changes in sensing or drug-release behaviour. Thus, the membrane effectively acts as a dynamic interface between the wound environment and the therapeutic system. The wound-dressing case therefore brings together many of the design principles developed throughout this review: tissue-matched mechanics, multilayer adhesion, transport regulation, electrical sensing, stimuli responsiveness, and long-term mechanical stability. Rather than simply protecting the wound, next-generation membranes can be designed as active interfaces that respond to changes in the mechanical and biochemical environment (Dong and Guo, 2021; Li et al., 2024).

## 9 Emerging directions, design framework and research gaps

The preceding sections demonstrate that the performance of polymeric membranes and thin films depends on the integration of tissue mechanics, polymer chemistry, fabrication-induced architecture, interfacial behaviour, transport, stimulus responsiveness, and long-term reliability. Future development, therefore, requires a transition from empirical material selection toward integrated, predictive, and physiologically validated design. Emerging approaches such as machine learning (ML), four-dimensional (4D) printing, organ-on-chip models, and biomimetic ultrathin membranes provide complementary tools for achieving this transition. At the same time, substantial challenges remain in linking material-scale properties to device-scale performance and ultimately to clinically relevant biological outcomes.

### 9.1 Machine-learning-assisted biomechanics-guided design

Machine learning provides an opportunity to address the multidimensional design space created by polymer composition, molecular architecture, fabrication parameters, membrane geometry, and physiological loading. Conventional trial-and-error optimisation becomes increasingly inefficient when several variables must be varied simultaneously. ML approaches, including Gaussian-process regression, neural-network surrogate models, and Bayesian optimisation, can instead establish relationships between processing conditions, material properties, and functional performance and identify promising regions of the design space (Raccuglia et al., 2016; Ramprasad et al., 2017). For polymeric membranes, this approach could integrate descriptors such as polymer chemistry, molecular weight, crosslink density, fibre orientation, pore size, thickness, and processing parameters with biomechanical outputs such as modulus, fatigue life, permeability, interfacial toughness, and strain sensitivity. The resulting models could then be coupled with finite-element simulations to predict membrane behaviour under application-specific physiological loading. Such integration would move the field from property prediction toward inverse design, in which the desired mechanical and functional response is specified first, and the material-architecture combination is subsequently identified. An important requirement for clinical translation is that ML models should be trained using physiologically relevant data, including wet-state properties, temperature, cyclic loading, degradation, protein adsorption, and interfacial behaviour, rather than relying predominantly on dry, room-temperature measurements. Li et al. highlights ML-assisted analysis of AFM force maps as an example of automated characterisation of heterogeneous polymer membranes and local elastic properties (Liu et al., 2023). More broadly, recent developments in ML for biomaterials and biomechanics support integrating data-driven prediction with mechanistic modelling, rather than treating ML as a replacement for physical understanding.

### 9.2 4D-printed adaptive membranes

Conventional additive manufacturing defines the three-dimensional geometry of a membrane or device during fabrication, whereas 4D printing introduces programmed changes in shape or mechanical state after fabrication. Shape-memory polymers and other stimuli-responsive networks can therefore be printed with spatially defined deformation trajectories, enabling a membrane to change its configuration in response to temperature, moisture, magnetic fields, or other stimuli. This capability is particularly attractive for biomedical membranes because physiological tissues are dynamic and patient anatomy can vary substantially. Multimaterial 4D printing has already demonstrated the ability to program spatially varying shape-memory responses (Ge et al., 2016). Bioresorbable 4D-printed systems may additionally allow mechanical properties to evolve alongside tissue regeneration, providing a potential strategy for addressing the temporal mechanical mismatch encountered in biodegradable devices (Miao et al., 2016). For membranes, however, the relevant design problem extends beyond shape transformation. Changes in geometry can modify membrane thickness, bending stiffness, pore structure, interfacial stresses, and transport pathways. Therefore, future 4D-printed membranes should be evaluated using coupled thermo-mechanical, fluid-structure, transport, and fatigue analyses. Reproducibility of programmed deformation, dimensional stability, sterilisation effects, degradation, and long-term actuation also require systematic investigation. An important translational gap is the absence of broadly established quality-control frameworks for time-dependent mechanical behaviour in 4D-printed biomedical membranes. Future standards will need to address not only the initial printed geometry but also the evolution of geometry and mechanical properties during activation, physiological ageing, and degradation.

### 9.3 Organ-on-chip validation

Organ-on-chip (OoC) platforms provide an important bridge between material-level testing and biological validation by reproducing physiologically relevant fluid flow, shear stress, cyclic strain, pressure, and tissue-material interactions in controlled *in-vitro* environments. Membranes within these systems can function simultaneously as cell-supporting substrates, selective barriers, transport interfaces, and mechanically actuated structures (Huh et al., 2010; Bhatia and Ingber, 2014). For biomechanics-guided membrane development, OoC platforms provide an opportunity to evaluate whether a membrane mechanically optimised at the material level actually produces the intended cellular response. For example, cardiovascular membranes can be subjected to cyclic pressure and flow while monitoring cardiomyocyte or endothelial responses; neural membranes can be evaluated under tissue-relevant deformation; and barrier membranes can be exposed to controlled fluid shear and transmembrane transport. Heart-on-chip systems are a particularly relevant example because they can combine cyclic mechanical loading, fluid flow, cellular mechanobiology, and material-tissue interactions on a single platform (Marsano et al., 2016). Current membrane-based OoC systems also demonstrate the importance of independently controlling membrane thickness, porosity, surface chemistry, stiffness, and permeability to reproduce organ-specific environments. The major opportunity is therefore to use OoC platforms as an intermediate validation stage between *in silico* design and animal/clinical studies. Such platforms could provide application-specific measurements of fatigue, transport, cell response, and biointerface stability under controlled physiological conditions. However, challenges remain in reproducing the full complexity of *in-vivo* loading, biological heterogeneity, immune responses, and long-term ageing.

### 9.4 Biomimetic ultrathin membranes

An emerging direction is the development of ultrathin polymeric films whose architecture and mechanical behaviour approach those of biological membranes and extracellular interfaces. Reducing membrane thickness provides a powerful means of lowering bending stiffness and improving conformability without necessarily requiring an extremely low bulk elastic modulus. This is particularly relevant for neural interfaces, wearable electronics, cell-contacting membranes, and other applications in which mechanical disturbance of the surrounding tissue must be minimized. Literature studies identify amphiphilic block-copolymer membranes and polymersomes as an extreme example of this design philosophy. Systems based on polymers such as PEG-b-PLA or PMPC-b-PDPA can form membranes with thicknesses in the nanometre range, providing highly compliant interfaces (Discher and Eisenberg, 2002). The concept can be extended beyond simply reducing thickness, and future biomimetic membranes could combine nanoscale thickness, controlled molecular organisation, selective transport, dynamic self-assembly, electrical functionality, and biological recognition. Such architectures could reproduce some of the structural principles of cellular membranes while incorporating synthetic functionalities unavailable in natural systems. The major challenge is maintaining sufficient mechanical robustness and defect tolerance at extreme thicknesses. At the nanoscale, surface forces, substrate constraints, residual stresses, defects, and interfacial adhesion can become dominant over bulk mechanical properties. Consequently, characterisation techniques capable of resolving nanoscale mechanical heterogeneity and long-term stability will be essential.

### 9.5 Toward an integrated biomechanics-guided design framework

The developments discussed suggest that the next-generation of polymeric membranes should be designed through an integrated multiscale workflow rather than by sequentially optimising individual properties. Figure 6 illustrates the framework established throughout this narrative review.

**FIGURE 6:**
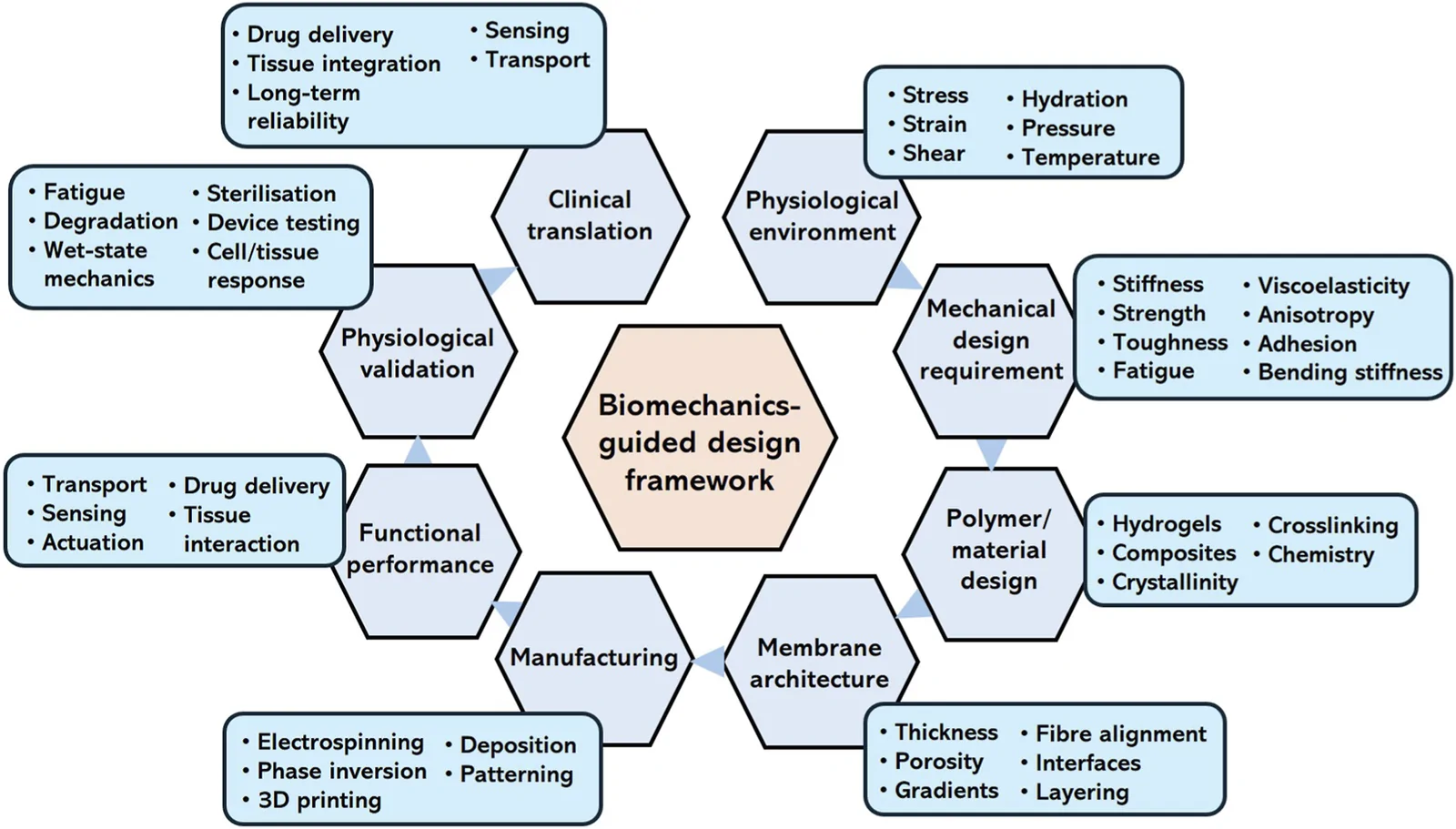
Biomechanics-guided design framework for polymeric membranes and thin films for medical devices.

At the first stage, the physiological environment defines the relevant loading mode, strain, stress, frequency, pressure, shear, and duration. These requirements are translated into target ranges for stiffness, extensibility, anisotropy, toughness, fatigue resistance, permeability, and interfacial adhesion. Polymer chemistry and molecular architecture are then selected to provide the required baseline properties, while fabrication determines the final fibre orientation, porosity, thickness, gradients, and multilayer architecture. The membrane should subsequently be evaluated under physiological rather than purely nominal conditions, including hydration, temperature, cyclic loading, protein exposure, degradation, and sterilisation. For smart membranes, stimulus-response characteristics should be evaluated together with mechanical stability. Finally, organ-specific biological models, including OoC platforms where appropriate, should be used to determine whether the engineered mechanical environment produces the intended cellular and functional responses. This framework changes the design question from “Which polymer has the required modulus?” to “Which material-architecture-interface combination produces the required mechanical and functional response throughout the intended service period?” This distinction is central to translating biomechanics-guided material design into reliable medical devices.

### 9.6 Limitations and translational challenges of emerging technologies

Despite their potential to advance the design of polymeric membranes and thin films for smart medical devices, machine learning, 4D printing, organ-on-chip systems, and biomimetic ultrathin membranes face several challenges that currently limit their translation into reliable biomedical applications (see Table 6).

**TABLE 6:** Emerging technologies, current limitations, and research priorities for biomechanics-guided polymeric membranes.

Emerging direction	Opportunity	Current limitation	Research priority
Machine learning	Rapid multi-parameter material/architecture optimisation	Limited physiologically relevant datasets	Physics-informed, wet-state and multi-objective ML
4D printing	Adaptive geometry and time-dependent mechanics	Limited long-term reliability and standards	Physiological fatigue, ageing and degradation validation
Organ-on-chip	Controlled mechanical and biological validation	Incomplete reproduction of *in-vivo* complexity	Standardised organ-specific mechanical environments
Biomimetic ultrathin membranes	Extreme conformability and low bending stiffness	Mechanical robustness and defect sensitivity	Nanoscale mechanics and long-term stability
Multilayer/graded architectures	Spatial control of mechanics and transport	Interface failure and manufacturing complexity	Predictive interfacial and gradient design
Smart multifunctional membranes	Integrated sensing, actuation and therapy	Competing mechanical/functional requirements	Multi-objective biomechanics-guided optimization

*Machine learning and computational prediction*: The predictive performance of machine-learning models depends on the availability, quality, and diversity of training data. Polymer datasets are often heterogeneous in terms of material composition, processing conditions, test protocols, and reported mechanical properties, which can limit comparisons across studies and the generalisability of trained models. Small datasets, inconsistent reporting, and limited representation of physiological loading conditions may further increase prediction uncertainty. Moreover, high predictive accuracy within a training dataset does not necessarily demonstrate reliable performance for new polymer formulations, membrane architectures, or biological environments. Experimental validation using independently prepared materials and application-relevant mechanical tests is therefore essential to establish model reliability.

*4D printing:* Although 4D printing enables time-dependent changes in shape or function in response to external stimuli, its performance depends on the reproducibility of material properties, printing parameters, and stimulus-responsive behaviour. Variations in print orientation, layer bonding, crosslinking, residual stresses, and environmental conditions can affect deformation, recovery, and dimensional stability. In addition, repeated actuation, prolonged exposure to physiological fluids, and changes in temperature or pH may alter the response over time. Demonstrating predictable and repeatable behaviour under physiologically relevant conditions remains important before these systems can be considered for long-term biomedical use.

*Organ-on-chip systems*: Organ-on-chip platforms can reproduce selected aspects of tissue structure, mechanical loading, and biochemical signalling, but their physiological relevance depends on how accurately these features represent the target tissue and its interactions with other biological systems. Differences in cell sources, culture conditions, device geometry, membrane properties, and fluid-flow conditions can lead to substantial variability between laboratories. Furthermore, simplified models may not fully capture immune responses, vascular interactions, tissue remodelling, or long-term biological adaptation. Standardised fabrication and testing protocols, appropriate biological controls, and validation against established *in vitro* or *in vivo* evidence are needed to improve reproducibility and confidence in their predictive value.

*Biomimetic ultrathin membranes*: Reducing membrane thickness can improve flexibility, conformability, and transport, but it also increases sensitivity to fabrication defects, handling damage, residual stress, and interfacial failure. Small variations in thickness, pore structure, surface chemistry, or substrate adhesion may produce meaningful differences in mechanical and functional performance. Long-term exposure to hydration, cyclic loading, sterilisation, and physiological degradation may further affect membrane integrity and device reliability. Consequently, characterisation should extend beyond initial mechanical properties to include defect assessment, interfacial stability, fatigue resistance, and performance following relevant environmental and ageing exposures.

Across these technologies, a common challenge is the gap between computational or conceptual demonstrations and experimentally verified device-level performance. Progress will require better reporting of material compositions and processing parameters, reproducible fabrication protocols, uncertainty assessment, and validation under application-specific mechanical, biological, and environmental conditions. Combining computational prediction with systematic experimental testing and, where appropriate, independent replication will be critical to establishing the reliability and translational potential of next-generation polymeric membranes and thin films.

## 10 Conclusion

Polymeric membranes and thin films serve as mechanically active components of a broad range of medical devices, and their successful design requires consideration of the mechanical environment in which they operate. A biomechanics-guided approach provides a framework for translating physiological loading conditions into material, architectural, and manufacturing requirements. The review highlights that mechanical compatibility extends beyond matching elastic modulus. Depending on the application, stiffness, bending behaviour, anisotropy, viscoelasticity, fracture resistance, fatigue performance, interfacial adhesion, and degradation behaviour may all influence device performance. Polymer chemistry provides a broad range of opportunities to tune these properties, while fabrication processes such as electrospinning, phase inversion, additive manufacturing, and thin-film deposition provide additional control over membrane architecture. The integration of mechanical behaviour with transport, sensing, actuation, and biological function is particularly important for next-generation smart membranes. However, laboratory measurements performed under simplified conditions may not adequately represent long-term behaviour in physiological environments. Wet-state testing, cyclic loading, physiological conditioning, interfacial characterisation, and device-level validation should therefore become integral components of membrane development. Emerging approaches based on machine learning, adaptive 4D printing, organ-on-chip systems, and biomimetic ultrathin membranes offer opportunities to shift from static material selection to adaptive, application-specific mechanical design. Future progress will depend on integrating these technologies with standardised mechanical characterisation and physiologically relevant validation. Ultimately, biomechanics-guided design should be viewed not as a single material-selection criterion but as an iterative process linking biological requirements, polymer chemistry, membrane architecture, manufacturing, mechanical performance, and clinical function.

## Nomenclature

AFM: atomic force microscopy
AM: additive manufacturing
AOA: amino-oleic acid
CVD: chemical vapour deposition
ePTFE: expanded polytetrafluoroethylene
HGO: holzapfel-gasser-ogden
IPN: interpenetrating network
MIO: metal-ion oxidation
ML: machine learning
OoC: organ-on-chip
PCL: polycaprolactone
PCU: polycarbonate-urethane
PDMS: polydimethylsiloxane
PDPA: poly(2-(diisopropylamino)ethyl methacrylate
PEG: poly(ethylene glycol)
PLA: poly(lactic acid)
PU: polyurethane
PES: polyethersulfone
PLGA: poly(lactic-co-glycolic acid)
PMPC: poly(2-methacryloyloxyethyl phosphorylcholine)
PNIPAM: poly(n-isopropylacrylamide)
PVD: physical vapour deposition
PVDF: poly(vinylidene fluoride)
PVP: polyvinylpyrrolidone
PSU: polysulfone
rGO: reduced graphene oxide
ROS: reactive oxygen species
TMP: transmembrane pressure
4D: four-dimensional
